# Toward Repurposing Ciclopirox as an Antibiotic against Drug-Resistant *Acinetobacter baumannii*, *Escherichia coli*, and *Klebsiella pneumoniae*

**DOI:** 10.1371/journal.pone.0069646

**Published:** 2013-07-23

**Authors:** Kimberly M. Carlson-Banning, Andrew Chou, Zhen Liu, Richard J. Hamill, Yongcheng Song, Lynn Zechiedrich

**Affiliations:** 1 Verna and Marrs McLean Department of Biochemistry and Molecular Biology, Baylor College of Medicine, Houston, Texas, United States of America; 2 Department of Molecular Virology and Microbiology, Baylor College of Medicine, Houston, Texas, United States of America; 3 Department of Medicine, Baylor College of Medicine, Houston, Texas, United States of America; 4 Department of Pharmacology, Baylor College of Medicine, Houston, Texas, United States of America; 5 Michael E. DeBakey VA Medical Center, Houston, Texas, United States of America; Monash University, Australia

## Abstract

Antibiotic-resistant infections caused by gram-negative bacteria are a major healthcare concern. Repurposing drugs circumvents the time and money limitations associated with developing new antimicrobial agents needed to combat these antibiotic-resistant infections. Here we identified the off-patent antifungal agent, ciclopirox, as a candidate to repurpose for antibiotic use. To test the efficacy of ciclopirox against antibiotic-resistant pathogens, we used a curated collection of *Acinetobacter baumannii*, *Escherichia coli*, and *Klebsiella pneumoniae* clinical isolates that are representative of known antibiotic resistance phenotypes. We found that ciclopirox, at 5–15 µg/ml concentrations, inhibited bacterial growth regardless of the antibiotic resistance status. At these same concentrations, ciclopirox reduced growth of *Pseudomonas aeruginosa* clinical isolates, but some of these pathogens required higher ciclopirox concentrations to completely block growth. To determine how ciclopirox inhibits bacterial growth, we performed an overexpression screen in *E. coli*. This screen revealed that *galE*, which encodes UDP-glucose 4-epimerase, rescued bacterial growth at otherwise restrictive ciclopirox concentrations. We found that ciclopirox does not inhibit epimerization of UDP-galactose by purified *E. coli* GalE; however, Δ*galU*, Δ*galE*, Δ*rfaI*, or Δ*rfaB* mutant strains all have lower ciclopirox minimum inhibitory concentrations than the parent strain. The *galU*, *galE*, *rfaI*, and *rfaB* genes all encode enzymes that use UDP-galactose or UDP-glucose for galactose metabolism and lipopolysaccharide (LPS) biosynthesis. Indeed, we found that ciclopirox altered LPS composition of an *E. coli* clinical isolate. Taken together, our data demonstrate that ciclopirox affects galactose metabolism and LPS biosynthesis, two pathways important for bacterial growth and virulence. The lack of any reported fungal resistance to ciclopirox in over twenty years of use in the clinic, its excellent safety profiles, novel target(s), and efficacy, make ciclopirox a promising potential antimicrobial agent to use against multidrug-resistant problematic gram-negative pathogens.

## Introduction

The World Health Organization lists antibiotic-resistant bacterial infections as an important public health problem [1]. In the U.S. alone, two million patients contract hospital-acquired infections, and 50–70% of these infections are antibiotic-resistant [2], resulting in the deaths of approximately 99,000 patients each year [3]. Longer hospital stays and increased morbidity and mortality as a consequence of antibiotic resistance translate to yearly estimated costs as high as $10 billion [3]. Gram-negative infections are particularly problematic and account for 47% of ventilator-associated pneumonias, 45% of urinary tract infections, and 70% of all intensive care unit infections [4]. If not appropriately treated, these infections can progress to sepsis and death.

Antibiotic-resistant gram-negative infections will continue to cause serious health problems because few of the antibiotics presently in development are effective against them [4], [5]. In addition, most new antibiotics are derivatives of existing drugs and, thus, have bacterial targets already under strong selection to develop resistance. The recent outbreak of carbapenem-resistant *Klebsiella pneumoniae* at the U.S. National Institutes of Health Clinical Center that caused six patient deaths illustrates how quickly these outbreaks spread and that vigilant precautions are needed for containment [6]. Identification of new antimicrobial agents, particularly those that affect novel targets, is needed to provide effective treatment options. Developing novel antimicrobial agents, however, usually takes a decade or more and costs millions of dollars. Repurposing already approved therapies for alternative uses saves both time and money [7]. Already such a strategy was used to find off-patent drugs to repurpose against antibiotic-resistant *Acinetobacter baumannii* [8].

To identify new antimicrobial agents with novel targets, our effort in targeting 1-deoxy-d-xylulose-5-phosphate reductoisomerase (DXR) yielded an N-hydroxypyridinone compound that shows broad-spectrum antibacterial activity [9]. A substructure based literature search led to the identification of ciclopirox, which has more potent antibacterial activity. Ciclopirox is an off-patent, topical antifungal drug developed almost forty years ago that is appealing to repurpose as an antibiotic because of its excellent safety profile. No fungal resistance has been identified in over twenty years of clinical use [10]. Indeed, others have suggested repurposing ciclopirox as an anti-human immunodeficiency virus drug [11], an agent to protect against mitochondrial damaged cells [12], and a way to enhance diabetic wound healing [13]. Additionally, ciclopirox is currently in a Phase I clinical trial for treatment of multiple myeloma [14], [15].

In spite of the multiple potential uses of ciclopirox, neither its drug target nor its mechanism of action is known. Genetic analyses in *Saccharomyces cerevisiae* and *Candida albicans* have been performed in attempts to understand how ciclopirox olamine functions, as this compound does not inhibit ergosterol biosynthesis like other antifungal agents [16]–[18]. In *S. cerevisiae*, a forward genetic screen identified fourteen mutants that were more susceptible to ciclopirox olamine. Mutations were identified in genes encoding proteins involved in DNA replication, DNA repair, cellular transport, oxidative stress, and signal transduction [16]. Results from *S. cerevisiae*, however, may not reflect the target of the drug because ciclopirox olamine only weakly inhibits *S. cerevisiae* growth [16]. A better experimental organism, the human pathogen, *C. albicans*, is susceptible to low µM ciclopirox olamine concentrations. Microarray analyses of ciclopirox olamine-treated *C. albicans* revealed gene expression level changes similar to those exhibited in iron-deprived conditions [17], [18]. Additionally, iron added to growth medium ameliorates ciclopirox olamine inhibition [17]–[19]. Together these data formed the basis for the model that ciclopirox olamine inhibits cells through general iron chelation, but that oxygen accessibility and additional iron-independent mechanisms may also influence ciclopirox olamine efficacy [10], [18].

Understanding how ciclopirox functions is important for uncovering additional repurposed clinical applications and could aid future ciclopirox derivatization. Here we demonstrate the effectiveness of ciclopirox against multidrug-resistant (MDR) *Escherichia coli*, *K. pneumoniae*, and *A. baumannii* clinical isolates. We show that ciclopirox affects the galactose salvage pathway, a novel mechanism of action for this drug.

## Results

### Ciclopirox Inhibits Growth of *E. coli* Clinical Isolates with a Range of Antibiotic Resistance Phenotypes

**Figure 1:**
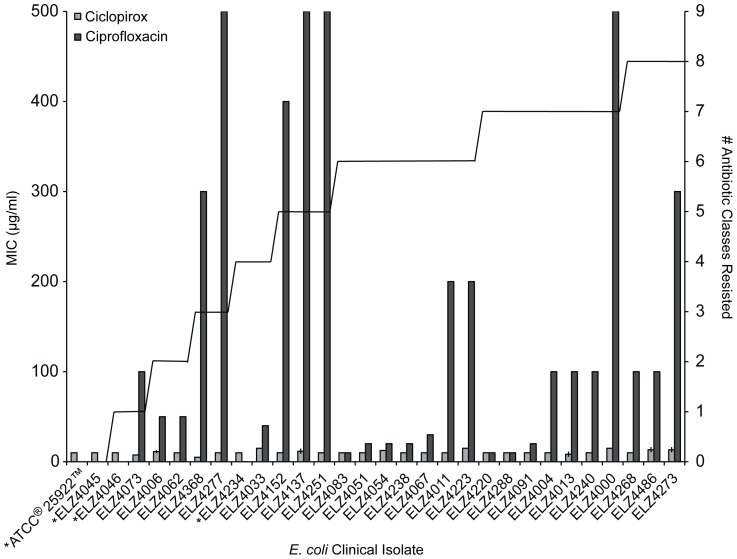
Ciclopirox and ciprofloxacin MICs for *E. coli* clinical isolates. The left *y*-axis is the average MIC in µg/ml for ciclopirox (light gray bar) and ciprofloxacin (dark gray bar). The clinical resistance breakpoint for ciprofloxacin is 4 µg/ml and an asterisk indicates isolates with MICs below this breakpoint. MIC measurements were repeated three times, and the average value and standard deviation is shown. The right *y*-axis denotes the number of antibiotic classes (aminoglycosides, carbapenems, cephalosporins, fluoroquinolones, monobactams, nitrofurans, penicillins, combination penicillins, and sulfamethozazole-trimethoprim) each isolate is resistant to, as indicated by the solid line.

Whereas ciclopirox previously was known to block growth of select gram-negative bacteria, its efficacy had not been tested against antibiotic-resistant bacteria [10], [20]. We took advantage of our curated collection of >4,000 *E. coli* clinical isolates with antimicrobial susceptibility patterns that range from fully sensitive to multidrug-resistant [21]–[23]. A list of these susceptibility profiles and the body sites from which these bacteria were isolated are included in Table S1 and Table S2. The ATCC® 25922™ *E. coli* strain was a control isolate that is drug-susceptible. For all experiments, we used ciclopirox without its conjugate salt, ethanolamine. Ethanolamine does not inhibit *E. coli* growth, but it can be metabolized as a nitrogen source and can serve as a signaling molecule, which could confound results [24]. We measured ciclopirox minimum inhibitory concentrations (MICs) for thirty non-clonal *E. coli* isolated from different patients and that represented the antibiotic resistance phenotypes in the collection. To verify the antibiotic resistance variation among these isolates, we simultaneously measured ciprofloxacin MICs, which varied widely as expected. Ciclopirox inhibited all of the tested strains at 5–15 µg/ml, independently of the antibiotic resistance status of the strains tested (Figure 1).

**Figure 2:**
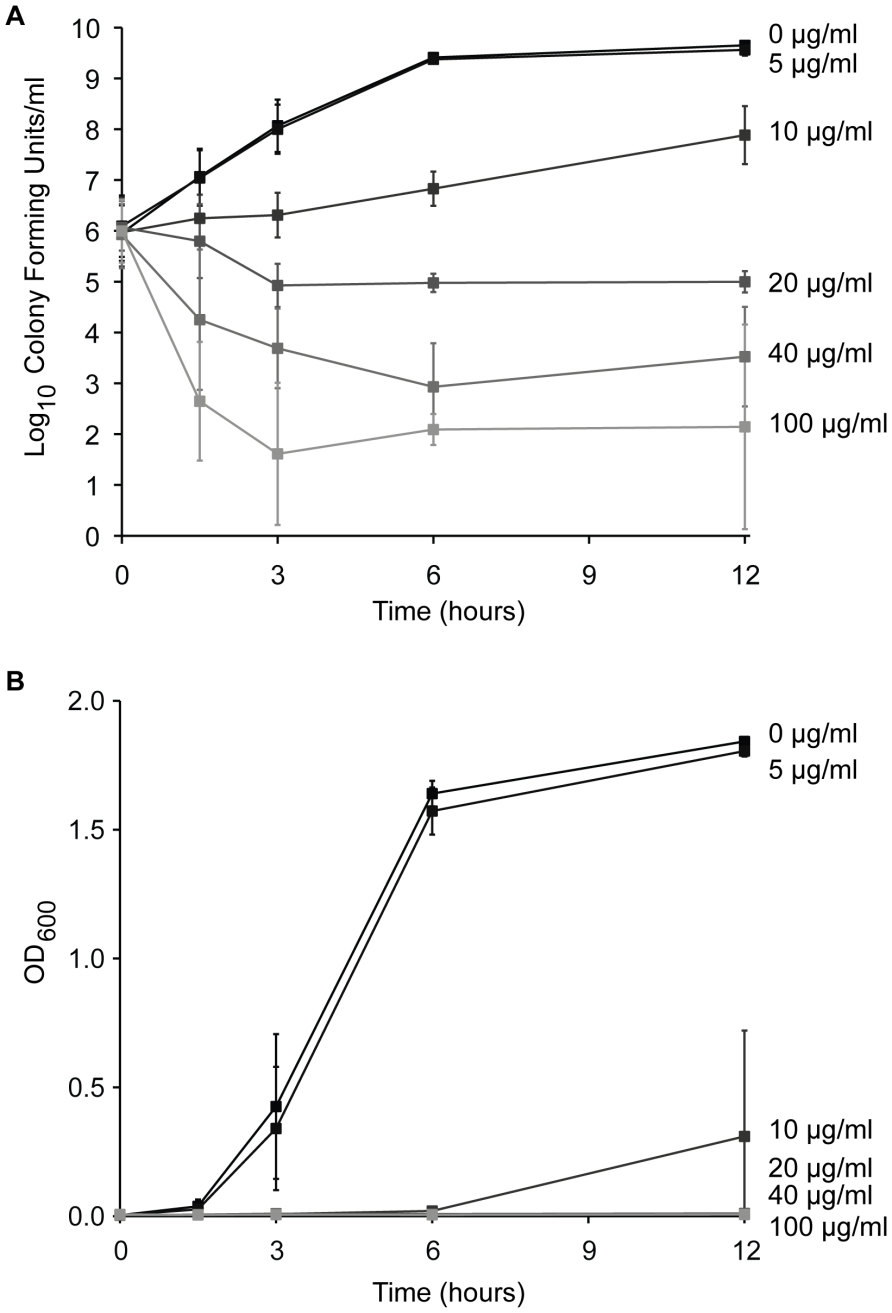
Time-kill and growth curves for *E. coli* with increasing ciclopirox concentrations. ATCC® 25922™ *E. coli* were grown with the indicated ciclopirox concentrations. (A) Cell densities were measured for three independent cultures by counting the number of colony forming units per 1 ml of culture for 12 hours. (B) The corresponding OD_600_ of growing cultures were measured over 12 hours. Error bars are the standard deviation from the mean.

To determine whether ciclopirox was bactericidal or bacteriostatic, we performed time-kill curves using the ATCC® 25922™ *E. coli* isolate. The ciclopirox MIC measured for this isolate was 10 mg/ml. We inoculated 10^6^ *E. coli* cells into medium with ciclopirox concentrations at one-half to 10x this MIC. The average colony forming units (CFUs) (Figure 2A) and the average OD_600_ (Figure 2B) are plotted for three independent experiments.

**Figure 3:**
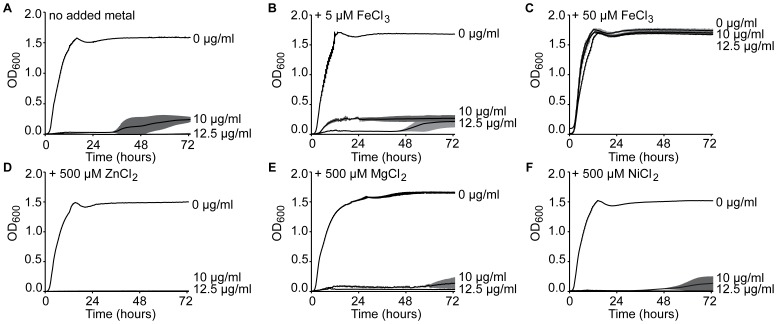
Effects of metals on exposure to ciclopirox for *E. coli*. Growth curves measured for three independent cultures of ATCC® 25922™ *E. coli* grown in (A) LB alone or (B) LB supplemented with 5 µM FeCl_3_, (C) 50 µM FeCl_3_, (D) 500 µM ZnCl_2_, (E) 500 µM MgCl_2,_ (F) or 500 µM NiCl_2_. Error bars are the standard deviation from the mean.

Cultures with either no drug or with 5 µg/ml ciclopirox both showed no growth inhibition (Figure 2A and Figure 2B). Compared to no drug, 10 µg/ml ciclopirox inhibited bacterial growth by two orders of magnitude after twelve hours, a drop that is consistent with bacteriostatic antibiotics (Figure 2A). The corresponding growth curves at the MIC (10 µg/ml) showed occasional delayed growth after six hours (Figure 2B). To test whether this growth resulted because of an *E. coli* coping strategy against ciclopirox, we re-inoculated the same culture in fresh medium supplemented with fresh 10 µg/ml ciclopirox and let cultures grow overnight. The culture growth was delayed as before, indicating that ciclopirox slowed growth at this concentration but this did not confer an added growth advantage to subsequent ciclopirox exposure. Only when previously ciclopirox-exposed cultures were inoculated into medium without ciclopirox could growth resume, again indicative of a bacteriostatic drug. It is unlikely that ciclopirox is unstable in the growth medium, as subsequent experiments showed that the drug inhibits bacterial growth for up to 72 hours (Figure 3), which exceeds the time indicated in Figure 2.

Ciclopirox concentrations of 20, 40, or 100 µg/ml, inhibited growth another order of magnitude following ciclopirox treatment. Bacterial death occurred within 90 minutes of exposure to ≥4x the ciclopirox MIC and within three hours following exposure to 2x the MIC, consistent with bactericidal antibiotics. By twelve hours following ciclopirox treatment, bacterial growth was further reduced with increasing drug dose. Therefore, at concentrations at or near the MIC, ciclopirox is a bacteriostatic drug, but at higher concentrations it is bactericidal.

### Iron Supplementation Prevents Ciclopirox-mediated *E. coli* Growth Inhibition

Iron chelation is a proposed mechanism of action for ciclopirox [17], [19]. Physiochemical studies showed that ciclopirox forms metal complexes with Mg^2+^, Ca^2+^, Cu^2+^, Fe^2+^, Zn^2+^, and Mn^2+^ and that these complexes display a wide range of water solubility and lipophilicity [25]. Researchers working with *C. albicans* demonstrated that medium supplemented with FeCl_3_ prevented ciclopirox olamine-mediated inhibition of fungal growth [17]–[19]. We tested whether addition of iron or other divalent cations could rescue *E. coli* exposed to ciclopirox. We measured growth of *E. coli* isolate ATCC® 25922™ in inhibitory ciclopirox concentrations supplemented with increasing concentrations of FeCl_3_, MgCl_2_, ZnCl_2_, or NiCl_2_. Compared to no added metals (Figure 3A), 5 µM FeCl_3_ allowed some bacterial growth (Figure 3B), while concentrations of 50 µM FeCl_3_ rescued bacterial growth in ciclopirox-treated cultures (Figure 3C). The other metals did not rescue growth, even at concentrations of 500 µM (Figure 3D, 3E, and 3F). These data show that ciclopirox inhibition can be ameliorated with high concentrations of iron. During a bacterial infection, however, the human host defense system actively sequesters free iron to limit pathogen growth [26]. Indeed, unbound ferric iron concentrations in plasma, lymph, and external secretions of milk and bronchial mucus have been reported to be ∼10^−18^ M, which is far below the 50 µM needed to rescue bacterial growth from ciclopirox inhibition [27]. While ciclopirox iron chelation would help attenuate infections, other iron-independent mechanisms of action may be involved or more important under physiological conditions.

### Effect of Ciclopirox on the Ability of *E. coli* to Survive Hydrogen Peroxide Exposure

**Figure 4:**
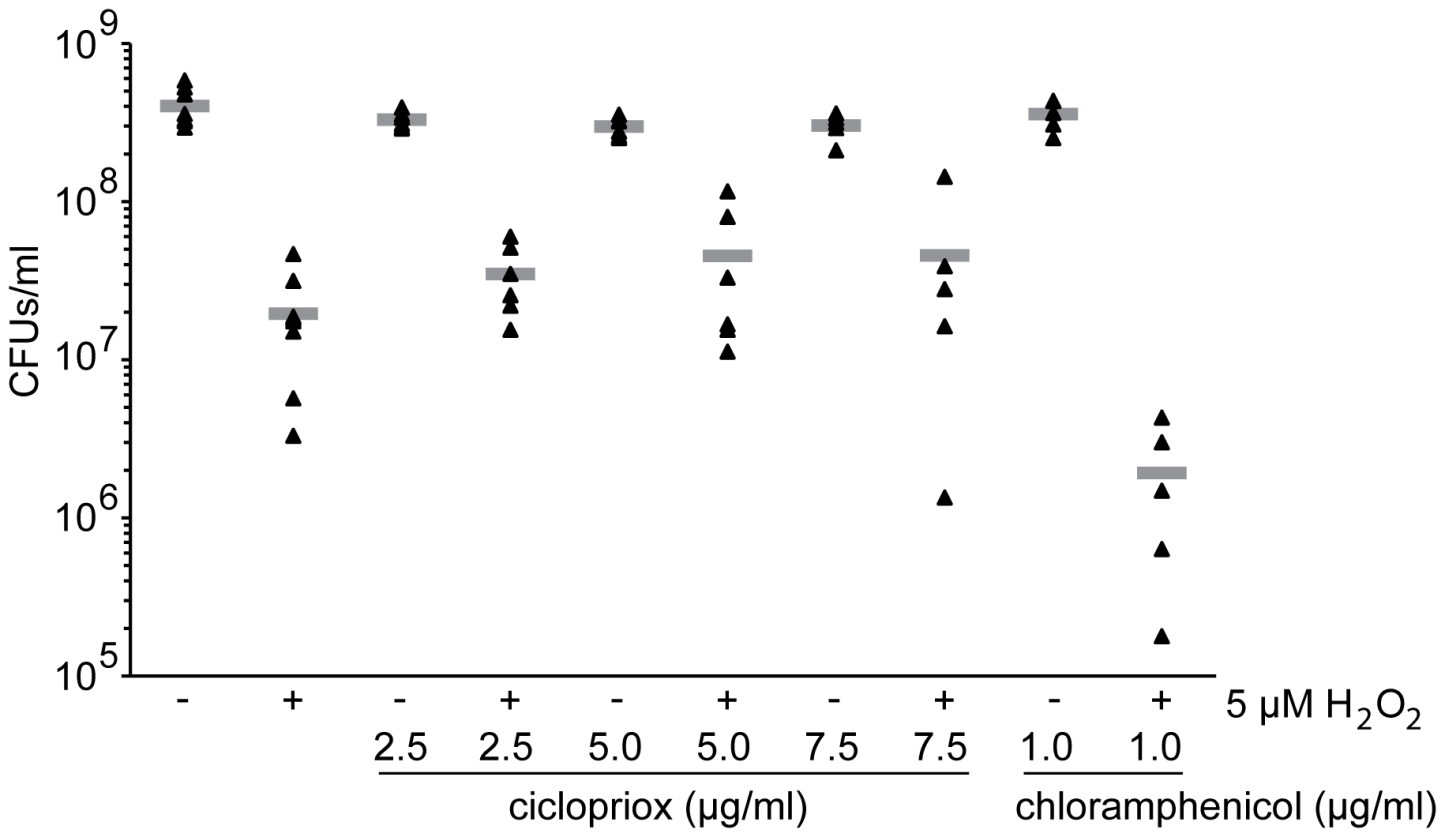
Effects of hydrogen peroxide exposure on bacterial response to ciclopirox. ATCC® 25922™ *E. coli* were grown to mid-logarithmic phase (OD_600_ = 0.4) and then subjected to either water or 5 µM H_2_O_2_ for 20 minutes. Colony forming units (CFUs) were measured per 1 ml of *E. coli* grown with either 0, 2.5, 5.0, or 7.5 µg/ml ciclopirox or 1.0 µg/ml chloramphenicol. Each triangle represents a result from an independent culture and the gray bar shows the average. Statistical significance was measured using Student’s t-test.

It was previously found that ciclopirox olamine sensitized *C. albicans* to H_2_O_2_ exposure [17], [18]. In addition, the expression of genes, like catalase, that detoxify reactive oxygen species (ROS) have been linked to ciclopirox olamine inhibition of *C. albicans* and for bactericidal antibiotic function [17], [18], [28]. We tested whether *E. coli* exposed to ciclopirox became sensitized to subsequent H_2_O_2_ exposure. *E. coli* isolate ATCC® 25922™ was grown with increasing sub-inhibitory concentrations of ciclopirox to mid-logarithmic phase and then exposed to either water or H_2_O_2_. Chloramphenicol served as a positive control because it sensitizes cells to H_2_O_2_ [29]. Whereas preincubation with 1 µg/ml chloramphenicol reduced CFUs following H_2_O_2_ exposure by more than 10-fold compared to water (Figure 4; p = 0.008), preincubation with ciclopirox had no effect. This lack of sensitization is consistent with the mechanism of action of some bacteriostatic antibiotics [28] and agrees with the above time-kill curve results at sub-inhibitory ciclopirox concentrations. These data contrast to the *C. albicans* data, most likely because ciclopirox is fungicidal at sub-inhibitory concentrations. However for bacterial growth, hydrogen peroxide exposure does not appear to synergize with sub-inhibitory ciclopirox concentrations.

### Overexpression of *galE* Rescues Ciclopirox Inhibition

Microarray analyses revealed that 25 of the 6,039 *C. albicans* genes were up-regulated and 21 were down-regulated with ciclopirox olamine incubation [18]. The majority of the up-regulated genes were involved in iron metabolism, and the rest included genes that encode Rbt5 glycosylphosphatidylinositol (GPI)-like proteins, transcription factors, an RNA binding protein, NADP-glutamate-dehydrogenase, superoxide dismutase Sod4, and two unknown proteins. The down-regulated genes included those encoding proteins involved with general stress responses, cell elongation, phosphate uptake, catalase, and many of unknown function. These microarray data indicated that some of these genes might encode targets of ciclopirox or proteins that might cause resistance to ciclopirox. Culturing *C. albicans* for six months with sub-inhibitory ciclopirox olamine concentrations, however, did not yield a resistant mutant [17].

**Figure 5:**
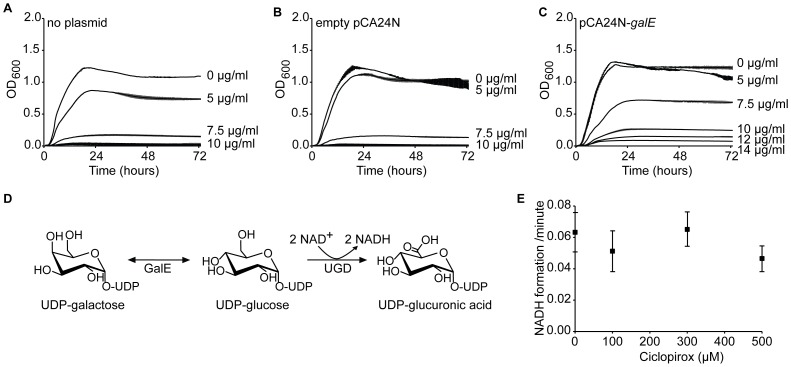
Effect of GalE on ciclopirox inhibition of bacterial growth. Growth curves for three independent cultures of TransforMax™ EC100™ Electrocompetent *E. coli* (A) without overexpression plasmid, (B) with empty plasmid pCA24N, or (C) with pCA24N-*galE* grown with the indicated ciclopirox concentrations. Error bars are the standard deviation from the mean. (D) Schematic representation of GalE epimerization of UDP-galactose to UDP-glucose coupled to the activity of UDP-glucose dehydrogenase (UGD). (E) Using the assay schematized in D, the average rate of NADH formation with or without ciclopirox was measured three independent times per ciclopirox concentration. Error bars are the standard deviation from the mean.

With more genetic tools available for *E. coli* than *C. albicans*, we reasoned that an overexpression suppression screen in *E. coli* would identify pathways affected by ciclopirox. To identify genes that, when overexpressed, rescued *E. coli* growth at otherwise restrictive ciclopirox concentrations, we transformed pools of plasmids from the ASKA (A Complete Set of *Escherichia coli* K-12 ORF Archive) pCA24N ORF library into TransforMax™ EC100™ Electrocompetent *E. coli*. Transformed cells were grown under selective ciclopirox concentrations of 7.5 µg/ml. 540 candidate transformants were streaked across agar containing a gradient of 0 to 18 µg/ml ciclopirox. MICs were then measured for 50 candidates that grew at higher ciclopirox concentrations than the other candidates. Only six of these transformants had ciclopirox MICs greater than the parent strain. The pCA24N plasmids from these six transformants were purified and the ORF was sequenced using previously described primers [30]. The purified plasmids were used to transform the parent strain, and the increase in ciclopirox MIC was confirmed. For all six candidates, the sequenced ORF was *galE*, which encodes UDP-glucose 4-epimerase. We confirmed that overexpression of *galE* rescued growth at previously restrictive ciclopirox concentrations (Figure 5A, 5B, and 5C). These data indentify GalE or the GalE pathway as a potential target of ciclopirox.

### Ciclopirox does not Inhibit Purified GalE

In *E. coli* and other bacteria, GalE epimerizes UDP-galactose and UDP-glucose. To test whether ciclopirox directly inhibits GalE, we purified 6xHis-GalE from *E. coli* DH5α cells transformed with the pCA24N-*galE* overexpression plasmid. Using previously described assays depicted in Figure 5D [31], [32], we coupled GalE activity to purified UDP-glucose dehydrogenase (UGH). UDP-galactose was used as a substrate for GalE. After GalE epimerizes UDP-galactose into UDP-glucose, UGH then converts UDP-glucose into UDP-glucuronic acid with the concomitant release of two molecules of NADH. The production of NADH is spectrophotometrically measured at 340 nm.

To assess whether ciclopirox could inhibit the epimerization of UDP-galactose, we measured NADH production in the presence of increasing ciclopirox concentrations (Figure 5E). For these experiments, ciclopirox was dissolved in 0.1 mM NaOH because we found that dimethyl sulfoxide (DMSO) inhibits GalE activity (Figure S1). For all reactions, the concentration of UDP-galactose used was 50 µM, which is below the published *K*_m_ (100–200 µM) for *E. coli* GalE. As a control, we ensured that NADH production was observed only when UDP-galactose was added; there was no spontaneous NADH formation. As an additional control, we verified that ciclopirox did not inhibit the coupled enzyme, UGD (Figure S2). GalE epimerization of UDP-galactose was not affected by ciclopirox, even at concentrations of 500 µM (Figure 5E). Although this assay does not address the possibility that ciclopirox may affect GalE epimerization of UDP-glucose into UDP-galactose, this reaction is less favored [33]. These data suggest that other targets in the GalE pathway are affected by ciclopirox, or that the nucleotide-sugars GalE produces help the bacteria cope with ciclopirox-induced cellular stresses.

### Effect of Mutations in the Galactose Salvage and Lipopolysaccharide Biosynthesis Pathways on Ciclopirox MICs

**Figure 6:**
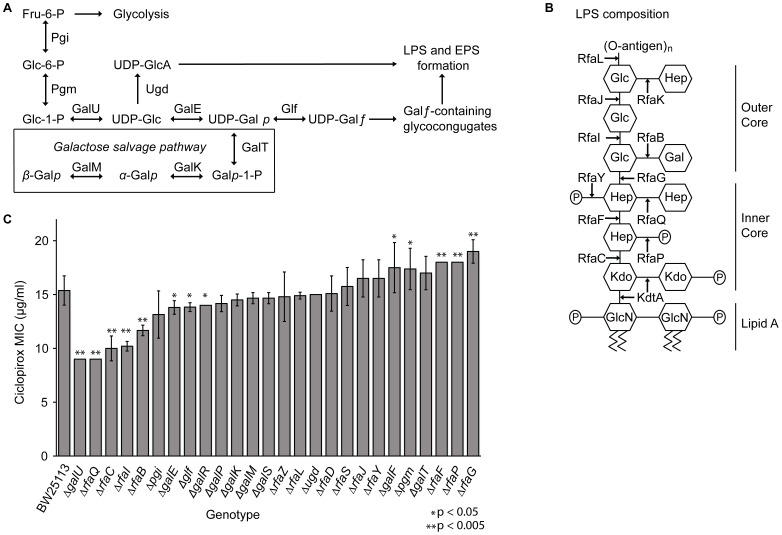
Effect of ciclopirox on strains involved in the galactose and lipopolysaccharide biosynthesis pathways. (A) Schematic representations of the galactose salvage pathway, (B) LPS biosynthesis pathway proteins for *E. coli* K-12 strain. Note the O-antigen is not present in K-12 strains, but is depicted for reference for where the O-antigen can attach. Abbreviations include fructose (Fru), glucose (Glc), galactose (Gal), heptose (Hep), *N*-acetylglucosamine (GlcN), 3-deoxy-d-*manno*-oct-2-ulopyranosonic acid (Kdo), and phosphate (P). (C) Ciclopirox MICs for Keio parent, BW25113, and single gene Keio deletion strains were measured in three independent cultures. These genes encode enzymes involved in the galactose metabolism and LPS biosynthesis pathways. Student’s t-test was used to assess significance.

Regulation of nucleotide-sugar concentrations is required for organisms to adjust to environmental stresses [34]–[36]. Depending on cellular needs, the galactose salvage pathway either metabolizes galactose for energy or uses galactose to build metabolic intermediates for lipopolysaccharide and exopolysaccharide construction (Figure 6A and 6B) [36]–[38]. When galactose or lactose is unavailable, GalE is required to synthesize UDP-galactose; however, when galactose is the sole carbon source, GalE synthesizes UDP-glucose, which is then converted to glucose-1-phosphate by GalU to be used in glycolysis [35], [36]. Thus, GalE is essential when *E. coli* is grown in galactose medium.

GalE is unique among the other members of the galactose salvage and LPS pathways. Even when grown in medium with other sugars, overexpression of most of the other galactose salvage pathway genes (*galK*, *galM*, *galP*, *galU*, *galR*, *galS*, and *galT*) are lethal for bacteria. Only overexpression of *glf* or *galE* is not lethal (Figure 6A) [30], [39]. The non-essential LPS biosynthesis genes, *rfaB*, *rfaC*, *rfaF*, *rfaG*, *rfaI*, *rfaL*, *rfaP*, *rfaQ*, *rfaY*, and *rfaZ* are also toxic when overexpressed in any sugar source, while the *rfaD*, *rfaJ*, or *rfaS* genes are not (Figure 6B) [30], [39]. However, in glucose medium, deletions of these same galactose metabolism or LPS biosynthesis genes are not lethal to bacteria.

Bacteria harboring deletions for proteins that promote cell survival upon ciclopirox exposure should make these bacteria more susceptible to the drug. Thus, ciclopirox MICs were measured in strains with deletion of genes known to be involved in the galactose salvage pathway (Δ*galE*, Δ*galK*, Δ*galM*, Δ*galU*, Δ*galT*, Δ*glf* and Δ*ugd*), the regulators of the galactose salvage pathway (Δ*galR* and Δ*galS*), the glycolysis pathway (Δ*pgi* and Δ*pgm*), or the lipopolysaccharide biosynthesis pathway (Δ*rfaB*, Δ*rfaC*, Δ*rfaD*, Δ*rfaF*, Δ*rfaG*, Δ*rfaI*, Δ*rfaJ*, Δ*rfaL*, Δ*rfaP*, Δ*rfaQ*, Δ*rfaS*, and Δ*rfaZ)*. Compared to the Keio parent strain, BW25113, 17/26 deletion mutants had altered average ciclopirox MICs; eight of these (Δ*galE*, Δ*galU*, Δ*galR*, Δ*glf*, Δ*rfaB*, Δ*rfaC*, Δ*rfaI*, and Δ*rfaQ*) were statistically significantly more susceptible to ciclopirox (Figure 6C; p = 0.05).

The Δ*rfaC*, Δ*rfaD*, Δ*rfaG*, Δ*rfaP*, Δ*rfaQ*, and Δ*rfaY* LPS pathway mutants had previously been shown to display enhanced sensitivity to other antibiotics compared to the parent BW25113 strain, but the galactose salvage pathway deletions were not affected by the tested antibiotics [40]. Therefore, whereas it was not surprising that the Δ*rfaC* and Δ*rfaQ* deletion mutant strains were more susceptible to ciclopirox, the other mutants that had increased sensitivity to ciclopirox were surprising. To determine whether the decrease in ciclopirox MIC in Δ*galE*, Δ*galU*, Δ*rfaB*, and Δ*rfaI* mutants was specific to ciclopirox or could be observed for other antibiotics, we measured ampicillin, aztreonam, chloramphenicol, and ciprofloxacin MICs (Table S3). Antibiotic MICs for Δ*rfaJ* mutants were also measured. Compared to BW25113, there were no significant susceptibility changes to ampicillin or ciprofloxacin. The Δ*rfaI* and Δ*rfaJ* strains were less susceptible to aztreonam, and the Δ*galE* and Δ*rfaB* strains were slightly more susceptible to chloramphenicol, but Δ*rfaJ* strains were less susceptible to chloramphenicol. These data suggest that the Δ*galE*, Δ*galU*, Δ*rfaB*, and Δ*rfaI* mutants are specifically more susceptible to ciclopirox, but not to other antibiotics tested.

Aside from RfaC and RfaQ, which are involved in synthesis of the inner LPS core, GalE, GalU, RfaB, and RfaI are all involved in synthesis of the outer LPS core, and all of these proteins either synthesize or utilize UDP-glucose or UDP-galactose. RfaJ is also involved in synthesis of the outer LPS and uses UDP-glucose, but Δ*rfaJ* mutants did not have altered ciclopirox susceptibility compared to the parental strain. These data suggest that LPS biosynthesis processes affected by ciclopirox could be dependent on the specific sugars or sugar linkages present.

Of the remaining deletion strains tested, five (Δ*galF*, Δ*pgm*, Δ*rfaF*, Δ*rfaP*, and Δ*rfaG*) were less susceptible to ciclopirox than the parental strain. The deletions mutants that were the least susceptible to ciclopirox, Δ*rfaF*, Δ*rfaG*, and Δ*rfaP*, encode proteins that synthesize the inner LPS core [41]. RfaF adds the first glucose group and RfaG adds heptose II to the developing inner LPS core, and deletions of these genes results in no outer core [41]. If no outer core is formed, then there is less demand for UDP-glucose or UDP-galactose. Instead of adding sugars to the inner core, RfaP phosphorylates heptose I and can affect RfaY and RfaQ functions [42]. While Δ*rfaP* mutants form both inner and outer LPS cores, the lack of phosphorylation affects overall membrane charge and surface hydrophobicity [41]. *E. coli* utilizes such membrane modification to resist antibiotics, such as polymyxin, and this resistance is clinically relevant [43], [44]. It is possible that changes in the membrane surface charge alters ciclopirox membrane permeability.

### Effect of Ciclopirox on LPS Formation

**Figure 7:**
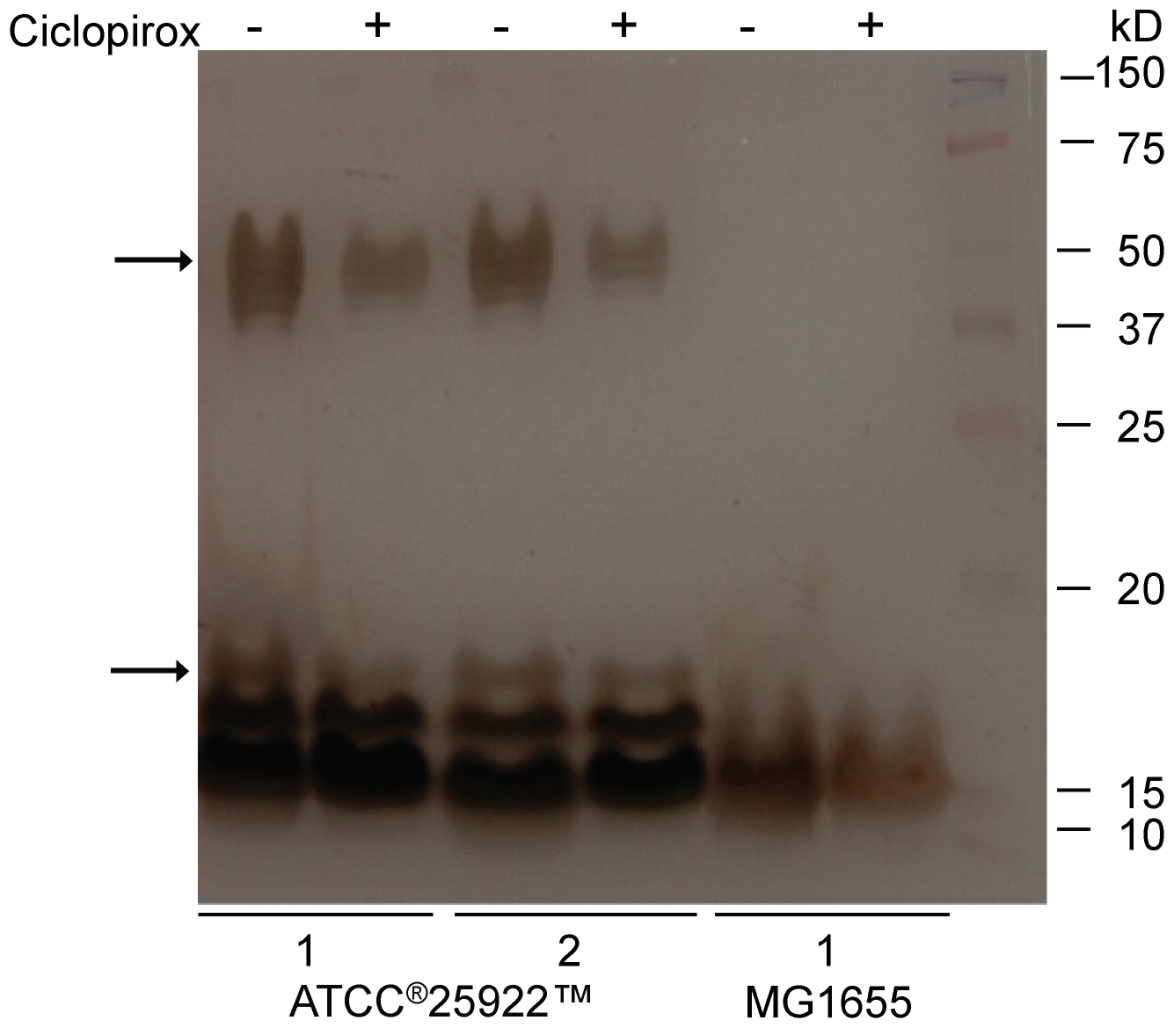
Effect of ciclopirox on *E. coli* LPS structure. LPS was purified from *E. coli* clinical isolate ATCC®25922™ or K-12 strain MG1655 that had been either incubated with 9 µg/ml ciclopirox as indicated. LPS was subjected to 12.5% Tris-glycine-SDS-PAGE. Lanes 1 and 2 are LPS from two independent LPS purifications. This result was repeated six times with the same results.

GalE is involved with LPS [45]–[47] and exopolysaccharide (EPS) formation [38], [48]. Studies in *C. albicans* have shown that ciclopirox alters the structure of cell membranes [17], [49], [50]. Indeed, 97% of ciclopirox administered to *C. albicans* was bound to cell membranes and organelles, with very little drug in the cytoplasm [50]. That we found ciclopirox MICs altered in strains deleted for genes responsible for LPS biosynthesis raises the possibility that ciclopirox alters LPS formation. To test this possibility, overnight cultures of the *E. coli* ATCC® 25922™ isolate or MG1655 strain were spread onto agar without or with a sub-inhibitory concentration of ciclopirox. After 24 hours, LPS was purified and subjected to SDS-PAGE as described [51] (Figure 7). Of the LPS bands characteristic of the ATCC® 25922™ isolate [52], ciclopirox reduced the concentrations of the highest molecular weight band (∼20 kDa) as well as the O-antigen bands (37–50 kDa), as indicated by the arrows (Figure 6). LPS isolated from MG1655, which lacks an O-antigen, was unchanged in the presence of ciclopirox. The LPS changes mediated by ciclopirox in the ATCC® 25922™ isolate were seen six independent times. These data may indicate a subtle mode of ciclopirox action: LPS is formed but with altered composition, perhaps as a consequence of which nucleotide-charged sugars are available.

### Ciclopirox Inhibits Growth of other Gram-negative Clinical Isolates with a Range of Antibiotic Resistance Phenotypes

Ciclopirox olamine inhibits the growth of *A. baumannii*, *K. pneumoniae*, *Proteus mirabilis*, *Pseudomonas aeruginosa*, and *E. coli*; however, the antibiotic resistance status for the tested bacteria was not indicated [8], [10], [20]. Given that ciclopirox inhibited growth of both antibiotic-susceptible and antibiotic-resistant *E. coli* clinical isolates, we tested it against additional problematic gram-negative pathogens [5].

We collected *A. baumannii*, *K. pneumoniae*, and *P. aeruginosa* clinical isolates from the Michael E. DeBakey VA Medical Center from November 2010 to August 2011. These bacterial species are often found in complex, polymicrobial infections. We therefore isolated the bacteria and identified them using 16S rRNA sequencing [53]. We then measured MICs for amikacin, ceftazidime, ciprofloxacin, imipenem, and trimethoprim/sulfamethoxazole (Table 1). The *K. pneumoniae* isolate ATCC®11296™, *A. baumannii* isolate ATCC®17978™, and *P. aeruginosa* isolate ATCC®27853™ were used as controls. All clinical isolates were resistant to ciprofloxacin except for ELZ7832 and ELZ8326. One *P. aeruginosa* isolate was resistant to amikacin and four had intermediate amikacin resistance. One *K. pneumoniae* isolate, seven *A. baumannii* isolates, and four *P. aeruginosa* isolates were resistant to imipenem, and six *K. pneumoniae*, five *A. baumannii*, and two *P. aeruginosa* isolates were resistant to ceftazidime. These clinical isolates thus harbor multiple antibiotic resistance mechanisms and are representative examples of gram-negative pathogens with few antibiotic options.

**Table 1:** Ciclopirox and other Antibiotic MICs in *K. pneumoniae*, *A. baumannii*, and *P. aeruginosa* clinical isolates.

		MIC (µg/ml)					
Species	Clinical Isolate	Ciclopirox	Ciprofloxacin	Ceftazidime	Imipenem	Amikacin	Trimethoprim/sulfamethoxazole
*K. pneumoniae*	ELZ7768	14–15	24–32	96–192	0.25	6	>32
*K. pneumoniae*	ELZ7813	15	32	96	4–6	3	3
*K. pneumoniae*	ELZ7870	5	16	96–128	0.19	6	>32
*K. pneumoniae*	ELZ7920	10	8–16	12	0.25	1.0–1.5	>32
*K. pneumoniae*	ELZ7832	13	2	24–64	0.75	48	>32
*K. pneumoniae*	ELZ7926	14	16–32	32–96	0.25–0.38	3	0.75
*K. pneumoniae*	ELZ8020	14	16	24	0.19	3	>32
*K. pneumoniae*	ATCC®11296™	12	0.25	0.25	0.25	2–3	>32
*A. baumannii*	ELZ7764	5–6	>32	>256	>32	12	>32
*A. baumannii*	ELZ7788	5–6	>32	>256	>32	12	>32
*A. baumannii*	ELZ7913	5–6	>32	8	>32	16	>32
*A. baumannii*	ELZ8002	6–7	>32	>256	>32	24	3
*A. baumannii*	ELZ8104	5–6	>32	8	>32	24	>32
*A. baumannii*	ELZ8319	5–7	>32	>256	>32	6	>32
*A. baumannii*	ELZ8326	5–6	0.25	3	0.25	0.5	>32
*A. baumannii*	ELZ8517	6–7	>32	96	>32	24	>32
*A. baumannii*	ATCC®17978™	7	0.25	4	0.38	2	>32
*P. aeruginosa*	ELZ7749	10	16–24	0.38	0.19	4	>32
*P. aeruginosa*	ELZ7762	15	8	256	16–24	4–6	2–3
*P. aeruginosa*	ELZ7776	30	8	1.5–2.0	1–2	6–8	24–>32
*P. aeruginosa*	ELZ7797	10	8	32–48	24	4–6	24–>32
*P. aeruginosa*	ELZ7820	>30	16	3	>32	96–192	>32
*P. aeruginosa*	ELZ7845	>30	8–16	4–6	>32	3–4	>32
*P. aeruginosa*	ELZ7847	30	16–24	1.0–1.5	1.0–1.5	8–12	4
*P. aeruginosa*	ELZ7918	>30	16	1.5	0.75	2–3	>32
*P. aeruginosa*	ATCC®27853™	>30	1	1.5	2–3	3	>32

Ciclopirox MICs in *K. pneumoniae* (n = 7) clinical isolates were 5 to 15 µg/ml. Strikingly, ciclopirox inhibited growth of the *A. baumannii* (n = 8) clinical isolates at 5 to 7 µg/ml, lower than ciclopirox olamine MICs previously reported [8]. This difference in MIC between ciclopirox and ciclopirox olamine could be attributable to the added ethanolamine salt. Whereas ciclopirox visibly reduced the growth of *P. aeruginosa* isolates, the MICs ranged from 10 to >30 µg/ml. In general, *P. aeruginosa* cultures that produce the siderophore, pyoverdin, are yellow-green and strains that produce the virulence factor, pyocyanin, are blue-green [54], [55]. The *P. aeruginosa* clinical isolates we tested were blue-green in liquid culture medium, indicative of pyocyanin production. The isolates treated with ciclopirox were yellow-green, indicating either a decrease in pyocyanin production or an upregulation of pyoverdin synthesis. To visualize this color change, we spread the *P. aeruginosa* isolate ATCC®27853™ on agar without or with 9 µg/ml ciclopirox. After 24 hours at 37°C, a lawn grew for both experimental conditions, but *P. aeruginosa* grown with ciclopirox was yellow-green (Figure S3). This ciclopirox-mediated color change could reflect important clinical implications. Cystic fibrosis patients infected with pyocyanin-producing *P. aeruginosa* strains suffer significant lung destruction [55]. It is possible that ciclopirox, while not inhibiting growth as effectively, attenuates *P. aeruginosa* virulence by affecting pyocyanin or pyoverdin synthesis. Ciclopirox, thus, may affect quorum sensing, which regulates pyocyanin synthesis [55], or iron utilization or iron transport, which regulates pyoverdin and siderophore synthesis [54}. Ciclopirox appears to have a complex mechanism of action, including both iron-dependent and iron-independent pathways, and this complexity may make ciclopirox a useful antibiotic against these gram-negative pathogens.

## Discussion

The lack of new antibiotic development has led to the use of toxic drugs, such as the polymyxins, when safer therapies are ineffective against treating antibiotic-resistant infections [5], [7]. The increase in antibiotic-resistant gram-negative pathogens is particularly problematic. *Enterobacteriaceae* producing extended-spectrum β-lactamases and carbapenemases cause nosocomial outbreaks that are spreading globally [6], [56]. MDR *A. baumannii* infections also have limited antibiotic therapeutic options available [7], [8] Repurposing already approved drugs for alternative use reduces the time and cost associated with antibiotic development. Ciclopirox is an excellent candidate to fulfill such a role.

The LPS and galactose salvaging pathways we found to be affected by ciclopirox represent promising targets for future drug development. *E. coli* galactose metabolism, also known as the Leloir pathway, is used for energy production, protein glycosylation, LPS biosynthesis, and virulence [36]. The amphibolic *galETKM* operon includes *galE* (UDP-glucose 4-epimerase), *galT* (galactose-1-phosphate uridylyltransferase), *galK* (galactokinase), and *galM* (galactose mutarotase) under control of two promoters, P1 and P2. During early to late logarithmic growth phase, 70% of the *galETKM* transcripts are synthesized from the P1 promoter and all four genes are made [35], [57]. However, upon transition from late logarithmic to early stationary phase, 70% of transcripts are from the P2 promoter, which predominately synthesizes *galE* [35], [57]. This differential promoter utilization and other posttranscriptional regulation of the *galETKM* operon using noncoding RNAs allows *E. coli* to either catabolize galactose or to generate the nucleotide-charged substrates, UDP-galactose or UDP-glucose, depending on cellular needs [35], [58].

When UDP-galactose accumulates, UTP and CTP concentrations are reduced and cell growth is halted; cells may even lyse depending on medium conditions [35]. Depletion of UTP also affects many *E. coli* promoters, including P1, that encode three uridine nucleotides. These uridine-rich promoters cause polymerase stuttering and subsequent depression of transcription [35]. Through induction of pyrimidine biosynthesis genes by regulators of the galactose pathway, the cell alleviates the stress of UTP depletion and restores UTP and CTP concentrations to rescue cell growth [35]. Therefore, disruptions in the regulation of the galactose pathway can be lethal to *E. coli* and bacteria have evolved multiple ways to regulate and control the pathway as a consequence.

In addition to regulating bacteria homeostasis, the galactose pathway has been directly linked to bacterial virulence and biofilm formation. In the enterohemorrhagic *E. coli* O157:H7 isolate, Δ*galETKM::aad-7* mutants have altered O-antigens, which reduces the ability of the mutant to colonize the intestines of rabbits, increases its sensitivity to host-derived antimicrobial polypeptides, and increases its sensitivity to bacteriophage P1 [46]. *Vibrio cholerae* O1 *galU* mutants also have reduced ability to colonize mouse small intestine [47]. A vaccine against *Salmonella enterica* serovar Typhi, the only live attenuated bacterial vaccine in the United States, is a *galE* mutant [46], [59]. In *Porphyromonas gingivalis, galE* mutants have shorter O-antigens, are more susceptible to antibiotics, and more readily form biofilms compared to the parental strain [60]. Conversely, GalE overexpression increases biofilm production in *Thermus thermophilus* HB27. Furthermore, *galE* and *galU* mutants in *V. cholerae* O1 had reduced biofilm-forming ability compared to wild-type strains [31], [47]. Together, these data connect GalE and the galactose salvage pathway to virulence and confirm that LPS and EPS biosynthesis pathways are potential targets for antimicrobial agents. In fact, novel GalE inhibitors are being actively derived and tested against *Trypanosoma brucei*, the causative agent for African sleeping sickness [32].

How sugars are metabolized and which sugars are predominantly used for energy vary widely in bacteria [61]. The model organisms, *E. coli* and *Bacillus subtilis* preferentially use glucose, metabolizing it through the Emden-Meyerhof-Parnas (EMP) pathway, and repress other sugar metabolism pathways when it is present [61], [62]. Other bacterial species, including *Pseudomonas* species, use glucose as a secondary carbon source and will catabolize glucose using the Entner-Doudoroff (ED) pathway [61], [62]. This differential use of glucose as a carbon source is a possible explanation for why *P. aeruginosa* clinical isolates had higher ciclopirox MICs than *E. coli* and *K. pneumoniae*. *Acinetobacter baylyi* also prefers glucose as a secondary carbon source [61], but the metabolic preference of *A. baumannii* is not yet clear, even though this knowledge could lead to ways to inhibit MDR *A. baumannii* [63]. Regardless of sugar preference, for pathogens to invade the host, they must compete with the normal microbiota for nutrients [61], [64]. Therefore, if ciclopirox inhibits the ability of a pathogen to utilize galactose, then it is likely that ciclopirox could attenuate virulence.

Our findings that ciclopirox affects galactose metabolism may explain why C. albicans is more susceptible to ciclopirox than S. cerevisiae [10], [16]. In yeast, the homologues of the bacterial galETKM operon are GAL10 (UDP-galactose-4-epimerase), GAL7 (Galactose-1-phossphate uridylyltransferase), and GAL1 (Galactokinase). Unlike E. coli GalE, the C. albicans GAL10 (CaGAL10) and the S. cerevisiae GAL10 (ScGAL10) homologues are larger with an added mutarose domain, similar to the bacterial GalM protein. GAL10 can also epimerize acetylated UDP-galactose and UDP-glucose [65]. Whereas ScGAL10 and CaGAL10 are both essential for growth when galactose is the sole carbon source, only CaGAL10 has additional functions to maintain cell-wall integrity and cell morphology, respond to oxidative stress, and proceed through normal hyphal morphogenesis in the absence of galactose [49].

The regulation of the GAL operon genes in *S. cerevisiae* also differs from *C. albicans* and other eukaryotic cells [49], [66], [67]. *S. cerevisiae* uses GAL80 to regulate GAL4, a transcriptional activator of the galactose operon [68]. Lacking a GAL80 ortholog and having a truncated GAL4 not involved in regulation of the GAL genes, *C. albicans* and other yeast instead use HGT4 to regulate GAL genes [66], [69]. The way *C. albicans* senses galactose directly affects cell morphology, virulence, and pathogenicity [70], [71]. These cell wall and morphology changes may help explain why *C. albicans* treated with ciclopirox exhibit reduced fungal adherence to buccal and vaginal epithelial cells [72].

Its effects on galactose metabolism or utilization may help explain why ciclopirox is effective against chronic myeloid leukemia and breast cancer cell lines compared to normal cells [15], [73]. Galactose and *N-*acetylglucosamine are required to form O-glycan core structures in eukaryotic cells [74]. O-glycan-containing structures have distinct terminal carbohydrates and these affect cell integrity and cell recognition, which should affect adherence to other cells [74]. In chronic myelogenous leukemia cells and in breast cancer MCF-7 cells, there is an increase in α3-sialytranserase activity, which produces shorter, sialylated O-glycans compared to normal myeloid and breast cells [75], [76]. Given the importance of O-glycosylation posttranslational modifications in the eukaryotic cell, we predict that ciclopirox may affect O-glycan structure in cancer cells. While normal cells would also express O-glycans, the amount of ciclopirox required to inhibit tumor growth is within achievable, non-toxic concentrations [14].

Just as iron supplementation alleviates ciclopirox inhibitory effects in *C. albicans*, FeCl_3_ supplementation rescued *E. coli* growth at previously restrictive ciclopirox concentrations. While ciclopirox is likely chelating iron in bacteria, *E. coli* also uses iron to signal modifications to the lipid A structure, an essential component of the LPS responsible for the toxic effects of gram-negative infections on the immune system [77]. The well-studied PmrA-PmrB two-component system is involved in this iron signaling pathway. Under high Fe^3+^ or low Mg^2+^ concentrations, PmrB activates PmrA to promote transcription of the *arn* operon. In conjunction with Ugd, the *arn* operon-encoded proteins synthesize 4-amino-4-deoxy-α-L-arabinose to incorporate it into the Lipid A structure, which alters the overall membrane charge [44], [78], [79]. PmrA also regulates expression of PmrC, which incorporates phosphoethanolamine into the Lipid A structure. This phosphoethanolamine modification is required for virulence of *Salmonella enterica* in mice and alters the membrane charge conferring antibiotic resistance to polymyxin B [80]. Therefore, under high iron concentrations, *E. coli* may modify its LPS to diminish the inhibitory effects of ciclopirox on bacterial growth.

Repurposing older drugs as broad-spectrum antibiotics or against specific problematic pathogens could be successful [7], [8]. Current topical formulations of ciclopirox olamine used to treat fungal skin infections and vaginal candidiasis penetrate deep into the skin and mucosal membranes without causing adverse systemic reactions [10]. It has already been shown that ciclopirox olamine applied topically to wounds induces angiogenesis and promotes faster wound healing [13]. Thus the excellent tolerability of ciclopirox and its unique mode(s) of action make it an attractive antibiotic to treat gram-negative pathogens, including those resistant to current antibiotic therapies.

## Materials and Methods

### Reagents

CaCl_2_, ethylenediamine tetraacetic acid (EDTA), glycerol, glycine, kanamycin, isopropyl β-D-1-thiogalactopyranoside (IPTG), MgCl_2_, NaCl, Na_2_HPO_4_, and Tris-HCl were from Fisher Scientific (Waltham, MA). β-NAD^+^, bovine UDP-glucose dehydrogenase, chloramphenicol, ciclopirox-olamine, DNAse I, imidazole, NH_4_Cl, MgSO_4_, UDP-galactose, UDP-glucose, dithiothreitol (DTT), and dimethyl sulfoxide (DMSO) were from Sigma-Aldrich (St. Louis, MO). Yeast extract, potassium monobasic phosphate, and potassium dibasic phosphate were from EMD Chemical (Gibbstown, NJ). EDTA-free inhibitor tablet and phenylmethylsulfonyl fluoride (PMSF) were from Roche (Mannheim, Germany). Taq DNA polymerase was from New England Biolabs (Ipswich, MA). Mueller-Hinton Broth, Bacto™ Tryptone, and Bacto™ Agar were from Becton, Dickinson and Company (Franklin Lakes, NJ). Ciclopirox was from AK Scientific Inc. (Union City, CA).

### Bacterial Strains, Clinical Isolates, and Growth Conditions

Strains used were BW25113 [39], TransforMax™ EC100™ Electrocompetent *E. coli* from Epicentre Biotechnologies (Madison WI), MG1655 and BL21(DE3) [81]. The *E. coli* clinical isolates used here were previously described [21]–[23] and were selected based on their antibiotic susceptibility or resistance to: aminoglycosides (amikacin, gentamicin and tobramycin), carbapenems (imipenem), cephalosporins (ceftriaxone, ceftazidime, cefotetan, cefepime, cefoxitin, cefotaxime and cefazolin), fluoroquinolones (ciprofloxacin and levofloxacin), monobactams (aztreonam), nitrofurans (nitrofurantoin), penicillins (ampicillin), combination penicillins (amoxicillin-clavulanic acid, piperacillin-tazobactam and ticarcillin-clavulanic acid), and sulfa drugs (sulfamethoxazole-trimethoprim). *K. pneumoniae* and *A. baumannii* clinical isolates were obtained from the Michael E. DeBakey VA Medical Center in Houston, Texas from November 2010 to August 2011. Strains and clinical isolates were grown in Mueller-Hinton (MH) or LB medium at 37°C. Chloramphenicol and kanamycin were used at 30 µg/ml, and ciclopirox concentrations were as indicated. LB agar had 1% tryptone, 0.5% yeast extract, 1% NaCl, and 15% agar. Plates containing agar with a gradient of ciclopirox were supplemented with 30 µg/ml chloramphenicol. The bottom layer had no ciclopirox and the top layer had 18 µg/ml ciclopirox [82].

### Bacterial Growth Curves

Overnight bacterial cultures were diluted 1∶100 in MH or LB medium and grown to an OD_600_ = 0.4 at 37°C. Cells were then centrifuged and resuspended in either MH or LB medium, as indicated, and diluted 1∶100 in medium containing various concentrations of ciclopirox. Bacterial growth in 100 well plates was also measured in MH and LB medium supplemented with various concentrations of MgCl_2_, FeCl_3_, ZnCl_2_, and NiCl_2_ and various ciclopirox concentrations, as indicated. Growth was measured under continuously shaking conditions, and OD_600_ was measured every half hour for three days using a BioscreenC machine and software (Oy Growth Curves Ab Ltd. Helsinki, Finland). Each ciclopirox and metal concentration was measured in triplicate and the average densities were plotted.

### MIC Determinations

Ciclopirox MICs were measured using microbroth dilution protocols done in accordance with Clinical Laboratory Standards Institute (CLSI) and measured in triplicate for concentrations that blocked visible bacterial growth [83]. Amikacin, ampicillin, aztreonam, ceftazidime, chloramphenicol, ciprofloxacin, imipenem, and trimethoprim/sulfamethoxazole MICs were measured by E-test® from AB bioMérieux (Marcy l’Etoile, France) and also by microbroth dilution when ciprofloxacin MICs were >32 µg/ml.

### Bacterial Time-kill Curves

Overnight ATCC® 25922™ *E. coli* cultures were diluted 1∶100 in LB medium and grown to an OD_600_ = 0.4 at 37°C. The cultures were then diluted 1∶100 in medium without or with 5, 10, 20, 40, or 100 µg/ml ciclopirox and grown shaking at 37°C. 1 ml of culture was removed upon inoculation and diluted. Diluted and undiluted cultures were spread onto LB agar plates and incubated overnight at 37°C. Over the next 12 hours, 1 ml of culture was removed, diluted, spread onto agar plates, and grown overnight at the times indicated. The number of colony forming units was then measured for each culture condition. Concomitantly, the OD_600_ was measured for each experimental condition described. All concentrations and times were measured for three independent overnight cultures and the values plotted are the average of those three experiments.

### Hydrogen Peroxide Assay

Overnight ATCC® 25922™ *E. coli* cultures were diluted 1∶100 in LB medium without or with 1 µg/ml chloramphenicol or 2.5, 5.0, or 7.5 µg/ml ciclopirox. All cultures were grown shaking to an OD_600_ = 0.4 at 37°C and then treated with 5 mM hydrogen peroxide or equal volumes of water for 20 minutes. 50 µg/ml catalase was added to quench each reaction. Diluted and undiluted cultures were spread onto LB agar plates and incubated overnight at 37°C. The number of colony forming units was then measured for each culture condition. Each experimental condition was repeated at least four times. The average CFUs/ml were plotted and significance was assessed using Student’s t-test.

### Overexpression Suppression Screen

TransforMax™ EC100™ Electrocompetent *E. coli* was transformed with pooled plasmids of the GFP^-^ ASKA ORF library [30] and spread on LB agar supplemented with chloramphenicol and 10 µM IPTG without or with 7.5 µg/ml ciclopirox. After overnight incubation at 37°C, colonies were streaked onto LB agar containing a ciclopirox gradient to identify transformants that could grow at concentrations restrictive to the TransforMax™ EC100™ Electrocompetent *E. coli*. pCA24N-based vectors harboring candidate genes were purified using the QIAprep® Spin MiniPrep Kit from QIAGEN® (Valencia, CA). The candidate gene was identified using PCR primers previously described [30]. 30 µl reactions using Taq polymerase were run for 25 cycles at 94°C for 5 minutes, 94°C for 30 seconds, 55°C for 30 seconds, 72°C for 2 minutes, and a final incubation at 72°C for 7 minutes. PCR products were purified using the QIAquick® PCR Purification Kit from QIAGEN® (Valencia, CA). Sequencing was performed by DeWalch Technologies (Houston, TX) and confirmed using genomic microbial BLAST [84], [85].

### GalE Purification

BL21(DE3) was transformed with pCA24N-*galE*. Overnight cultures were used to inoculate 1 L of LB supplemented with chloramphenicol. Cells were grown shaking at 37°C to OD_600_ = 0.4 and 1 mM IPTG was added for 2 hours. Cells were then centrifuged at 6,000 rpm for 15 minutes and pellets were frozen at −80°C overnight. Pellets were resuspended in lysis buffer (25 mM potassium phosphate, pH 7.5, 500 mM NaCl, 10 mM imidazole, 1 EDTA-free inhibitor tablet, 2 mg/ml DNAse I, and 37.5 µM MgCl_2_). On ice, cells were sonicated using a 1/8-inch probe sonicator Model 60 Sonic Dismembrator from Fisher Scientific (Waltham, MA) for five 30-second intervals over 10 minutes at 14 Watts. Lysates were centrifuged and filtered using a Corning 0.45 µm sterile syringe filter (Corning, NY). All subsequent purification was performed at 4°C. For 1 hour, the lysate was mixed with TALON Metal Affinity Resin beads from Clontech Laboratories, Inc. (Mountain View, CA). Beads were washed in 40 ml of 25 mM potassium phosphate pH 7.5, 500 mM NaCl, 10 mM imidazole, and 1 EDTA-free inhibitor tablet. Protein was eluted in four 5 ml fractions of 25 mM potassium phosphate, pH 7.5, 500 mM NaCl, 250 mM imidazole, and 1 EDTA-free inhibitor tablet. Protein concentrations were determined using a Coomassie (Bradford) Protein Assay Kit from Thermo Fisher Scientific Inc. (Rockford, IL). Fractions were analyzed by 12% sodium dodecyl sulfate polyacrylamide gel electrophoresis (SDS-PAGE), and proteins were visualized with Coomassie Blue staining. Fractions with purified GalE were dialyzed in two 1 L exchanges of 20 mM Tris-HCl, pH 8.0, 1 mM EDTA pH 8.0, 1 mM PMSF, and 1 mM DTT. The enzyme was used immediately.

### GalE and UDP-glucose Dehydrogenase Enzymatic Assay

GalE enzymatic activity was assessed as previously published [31], [32]. In brief, 1 µg purified GalE was added to an overall reaction volume of 500 µl containing 100 mM glycine, pH 8.9, 1 mM β-NAD^+^, 8.3 mM MgCl_2_, and 40 mU bovine UDP-glucose dehydrogenase. NADH formation was monitored spectrophotometrically at 340 nm for 5 minutes following the addition of 4 mM UDP-galactose. Enzyme function was assessed either in the absence of or with increasing concentrations of ciclopirox. To assess whether ciclopirox affected UDP-glucose dehydrogenase, 40 mU of UDP-glucose dehydrogenase was used under the same conditions. NADH formation was monitored following the addition of 4 mM UDP-glucose.

### LPS Analysis

LPS constituents were analyzed by SDS-PAGE as described [51]. Briefly, *E. coli* was grown overnight on LB agar either without or with 9 µg/ml ciclopirox. The bacteria were suspended in phosphate-buffered saline (pH 7.2) and adjusted to an OD_600_ of 2. Cells were lysed and digested with DNaseI and proteinase K. Digested proteins were extracted with phenol. Trace amounts of phenol were removed with saturated ethyl ether. LPS samples were stored at −20°C until analysis by 12.5% Tris-Glycine-SDS-PAGE. Bands were visualized by silver staining. Bio-Rad Precision Plus Protein™ Standards Kaleidoscope™ ladder was the molecular weight marker.

### 16S rRNA Sequencing of Gram-negative Clinical Isolates

To identify specific gram-negative pathogens in complex polymicrobial clinical isolates, we isolated single colonies and extracted their genomic DNA using the Promega Wizard® Genomic Purification Kit. This DNA was used as the template to amplify the 16S rRNA gene. PCR conditions were the same as those outlined for amplification of ASKA library plasmids above. The forward primer 27f-MP (5′-AGRGTTTGATCMTGGCTCAG) and reverse primer 1492r-MP (5′-TACGGYTACCTTGTTAYGACTT) have been previously used for species identification [53]. PCR product quality was validated by visualization with gel electrophoresis on a 1% agarose gel. PCR products were purified using the QIAquick® PCR Purification Kit and the 16S rRNA products were sequenced by DeWalch Technologies (Houston, TX). Sequence identity was confirmed using genomic microbial BLAST and the Ribosomal Database Project (RDP) [84]–[86].

## Supporting Information

Figure S1 **Effect of DMSO on GalE epimerization.** (A) Schematic representation of GalE epimerization of UDP-galactose to UDP-glucose coupled to the activity of UDP-glucose dehydrogenase (UGD). (B) Using the assay schematized in A, the average rate of NADH formation with or without DMSO was measured three independent times per DMSO percentage. Error bars are the standard deviation from the mean.
(TIF)

Figure S2 **Effect of ciclopirox on UDP-glucose dehydrogenase.** (A) Schematic representation of UDP-glucose dehydrogenase (UGD) activity converting UDP-glucose into UDP-glucuronic acid. (B) Using the assay schematized in A, the average rate of NADH formation with or without ciclopirox was measured three independent times per ciclopirox percentage. Error bars are the standard deviation from the mean.
(TIF)

Figure S3 **Ciclopirox effects on** ***P. aeruginosa*** **growth.** Mid-logarithmic cultures (O.D. = 0.4) of ATCC®27853™ *P. aeruginosa* cultures were spread onto LB agar without (left) or with 9 µg/ml ciclopirox (middle) and grown at 37°C. After 24 hours, plates were imaged.
(TIF)

Table S1 **Antibiotic susceptibility status for fluoroquinolone-resistant clinical isolates.**
(DOCX)

Table S2 **Patient demographics and clinical isolate culture sites.**
(DOCX)

Table S3 **Antibiotics MICs in galactose pathway deletion strains.**
(DOCX)

## References

[1] Collignon P, Powers JH, Chiller TM, Aidara-Kane A, Aarestrup FM (2009) World Health Organization ranking of antimicrobials according to their importance in human medicine: A critical step for developing risk management strategies for the use of antimicrobials in food production animals. Clin Infect Dis 49: 132–141. 19489713 10.1086/599374

[2] Mauldin PD, Salgado CD, Hansen IS, Durup DT, Bosso JA (2010) Attributable hospital cost and length of stay associated with health care-associated infections caused by antibiotic-resistant gram-negative bacteria. Antimicrob Agents Chemother 54: 109–115. 19841152 10.1128/AAC.01041-09 PMC2798544

[3] Peleg AY, Hooper DC (2010) Hospital-acquired infections due to gram-negative bacteria. N Engl J Med 362: 1804–1813. 20463340 10.1056/NEJMra0904124 PMC3107499

[4] Hidron AI, Edwards JR, Patel J, Horan TC, Sievert DM, et al (2008) NHSN annual update: antimicrobial-resistant pathogens associated with healthcare-associated infections: annual summary of data reported to the National Healthcare Safety Network at the Centers for Disease Control and Prevention, 2006–2007. Infect Control Hosp Epidemiol 29: 996–1011. 18947320 10.1086/591861

[5] Boucher HW, Talbot GH, Bradley JS, Edwards JE, Gilbert D, et al (2009) Bad bugs, no drugs: no ESKAPE! An update from the Infectious Diseases Society of America. Clin Infect Dis 48: 1–12. 19035777 10.1086/595011

[6] Snitkin ES, Zelazny AM, Thomas PJ, Stock F, Henderson DK, et al (2012) Tracking a hospital outbreak of carbapenem-resistant *Klebsiella pneumoniae* with whole-genome sequencing. Sci Transl Med 4: 148ra116. 10.1126/scitranslmed.3004129 PMC3521604 22914622

[7] Fischbach MA (2009) Antibiotics for Emerging Pathogens. Science 325: 1089–1093. 19713519 10.1126/science.1176667 PMC2802854

[8] Chopra S, Torres-Ortiz M, Hokama L, Madrid P, Tanga M, et al (2010) Repurposing FDA-approved drugs to combat drug-resistant *Acinetobacter baumannii* . J Antimicrob Chemother 65: 2598–2601. 20861141 10.1093/jac/dkq353

[9] Deng L, Sundriyal S, Rubio V, Shi Z, Song Y (2009) Coordination chemistry based approach to lipophilic inhibitors of 1-deoxy-D-xylulose-5-phosphate reductoisomerase. J Med Chem 52: 6539–6542. 19888756 10.1021/jm9012592

[10] Subissi A, Monti D, Togni G, Mailland F (2010) Ciclopirox: recent nonclinical and clinical data relevant to its use as a topical antimycotic agent. Drugs 70: 2133–2152. 20964457 10.2165/11538110-000000000-00000

[11] Hoque M, Hanauske-Abel HM, Palumbo P, Saxena D, D’Alliessi Gandolfi D, et al (2009) Inhibition of HIV-1 gene expression by Ciclopirox and Deferiprone, drugs that prevent hypusination of eukaryotic initiation factor 5A. Retrovirology 6: 90. 19825182 10.1186/1742-4690-6-90 PMC2770518

[12] Lee SJ, Jin Y, Yoon HY, Choi B-O, Kim HC, et al (2005) Ciclopirox protects mitochondria from hydrogen peroxide toxicity. Br J Pharmacol 145: 469–476. 15806112 10.1038/sj.bjp.0706206 PMC1576158

[13] Ko SH, Nauta A, Morrison SD, Zhou H, Zimmermann A, et al (2011) Antimycotic ciclopirox olamine in the diabetic environment promotes angiogenesis and enhances wound healing. PloS One 6: e27844. 22125629 10.1371/journal.pone.0027844 PMC3220686

[14] Weir SJ, Patton L, Castle K, Rajewski L, Kasper J, et al (2011) The repositioning of the anti-fungal agent ciclopirox olamine as a novel therapeutic agent for the treatment of haematologic malignancy. J Clin Pharm Ther 36: 128–134. 21366640 10.1111/j.1365-2710.2010.01172.x

[15] Eberhard Y, Mcdermott SP, Wang X, Gronda M, Venugopal A, et al (2009) Chelation of intracellular iron with the antifungal agent ciclopirox olamine induces cell death in leukemia and myeloma cells. Blood 114: 3064–3073. 19589922 10.1182/blood-2009-03-209965

[16] Leem S-H, Park J-E, Kim I-S, Chae J-Y, Sugino A, et al (2003) The possible mechanism of action of ciclopirox olamine in the yeast *Saccharomyces cerevisiae* . Mol Cells 15: 55–61. 12661761

[17] Niewerth M, Kunze D, Seibold M, Schaller M, Korting HC, et al (2003) Ciclopirox Olamine Treatment Affects the Expression Pattern of *Candida albicans* Genes Encoding Virulence Factors, Iron Metabolism Proteins, and Drug Resistance Factors. Antimicrob Agents Chemother 47: 1805–1817. 12760852 10.1128/AAC.47.6.1805-1817.2003 PMC155814

[18] Sigle H-C, Thewes S, Niewerth M, Korting HC, Schäfer-Korting M, et al (2005) Oxygen accessibility and iron levels are critical factors for the antifungal action of ciclopirox against *Candida albicans* . J Antimicrob Chemother 55: 663–673. 15790671 10.1093/jac/dki089

[19] Dittmar W, Lohaus G (1973) HOE 296, a new antimycotic compound with a broad antimicrobial spectrum. Laboratory results. Arzneimittelforschung 23: 670–674. 4197001

[20] Jue SG, Dawson GW, Brogden RN (1985) Ciclopirox olamine 1% cream. A preliminary review of its antimicrobial activity and therapeutic use. Drugs 29: 330–341. 3158508 10.2165/00003495-198529040-00002

[21] Becnel Boyd L, Maynard MJ, Morgan-Linnell SK, Horton LB, Sucgang R, et al (2009) Relationships among ciprofloxacin, gatifloxacin, levofloxacin, and norfloxacin MICs for fluoroquinolone-resistant *Escherichia coli* clinical isolates. Antimicrob Agents Chemother 53: 229–234. 18838594 10.1128/AAC.00722-08 PMC2612140

[22] Morgan-Linnell SK, Becnel Boyd L, Steffen D, Zechiedrich L (2009) Mechanisms accounting for fluoroquinolone resistance in *Escherichia coli* clinical isolates. Antimicrob Agents Chemother 53: 235–241. 18838592 10.1128/AAC.00665-08 PMC2612180

[23] Swick MC, Morgan-Linnell SK, Carlson KM, Zechiedrich L (2011) Expression of multidrug efflux pump genes *acrAB-tolC*, *mdfA*, and *norE* in *Escherichia coli* clinical isolates as a function of fluoroquinolone and multidrug resistance. Antimicrob Agents Chemother 55: 921–924. 21098250 10.1128/AAC.00996-10 PMC3028778

[24] Garsin DA (2012) Ethanolamine : A Signal to Commence a Host-Associated Lifestyle? MBio 3: 1–4 doi:10.1128/mBio.00172-12 10.1128/mBio.00172-12 PMC3398539 22761393

[25] Tarawneh RT, Hamdan II, Bani-Jaber A, Darwish RM (2005) Physicochemical studies on Ciclopirox olamine complexes with divalent metal ions. Int J Pharm 289: 179–187. 15652210 10.1016/j.ijpharm.2004.11.009

[26] Weinberg ED (2009) Iron availability and infection. Biochim Biophys Acta 1790: 600–605. 18675317 10.1016/j.bbagen.2008.07.002

[27] Bullen Rogers, H.J Griffiths, E JJ (1978) Role of iron in bacterial infection. Arber W, Henle W, Hofschneider PH, Humphrey JH, Klein J, et al., editors Springer Berlin Heidelberg. 1–35.

[28] Dwyer DJ, Kohanski M a, Collins JJ (2009) Role of reactive oxygen species in antibiotic action and resistance. Curr Opin Microbiol 12: 482–489. 19647477 10.1016/j.mib.2009.06.018 PMC2761529

[29] Loui C, Chang AC, Lu S (2009) Role of the ArcAB two-component system in the resistance of *Escherichia coli* to reactive oxygen stress. BMC Microbiol 9: 183. 19715602 10.1186/1471-2180-9-183 PMC2748088

[30] Kitagawa M, Ara T, Arifuzzaman M, Ioka-Nakamichi T, Inamoto E, et al (2005) Complete set of ORF clones of *Escherichia coli* ASKA library (a complete set of *E. coli* K-12 ORF archive): unique resources for biological research. DNA research : an international journal for rapid publication of reports on genes and genomes. DNA Res 12: 291–299. 16769691 10.1093/dnares/dsi012

[31] Niou Y-K, Wu W-L, Lin L-C, Yu M-S, Shu H-Y, et al (2009) Role of *galE* on biofilm formation by *Thermus* spp. Biochem Biophys Res Commun 390: 313–318. 19800315 10.1016/j.bbrc.2009.09.120

[32] Urbaniak MD, Tabudravu JN, Msaki A, Matera KM, Brenk R, et al (2006) Identification of novel inhibitors of UDP-Glc 4′-epimerase, a validated drug target for african sleeping sickness. Bioorg Med Chem Lett 16: 5744–5747. 16962325 10.1016/j.bmcl.2006.08.091

[33] Chen X, Kowal P, Hamad S, Fan H, Wang PG (1999) Cloning, expression and characterization of a UDP-galactose 4-epimerase from *Escherichia coli* . Biotechnol Lett 21: 1131–1135.

[34] Sousa Feliciano, Joana R., and Jorge H. Leitão SA (2011) Biotechnology of Biopolymers. Activated Sugar Precursors: Biosynthetic Pathways and Biological Roles of an Important Class of Intermediate Metabolites in Bacteria. In: Magdy Elnashar, editor. Biotechnology of Biopolymers. InTech. 257–274.

[35] Lee SJ, Trostel A, Le P, Harinarayanan R, Fitzgerald PC, et al (2009) Cellular stress created by intermediary metabolite imbalances. Proc Natl Acad Sci U S A 106: 19515–19520. 19887636 10.1073/pnas.0910586106 PMC2780772

[36] Csiszovszki Z, Krishna S, Orosz L (2011) Structure and Function of the d-Galactose Network in Enterobacteria. MBio 2: 1–8 doi:10.1128/mBio.00053-11 10.1128/mBio.00053-11 PMC3119520 21712421

[37] Raetz CRH, Whitfield C (2002) Lipopolysaccharide endotoxins. Annu Rev Biochem 71: 635–700. 12045108 10.1146/annurev.biochem.71.110601.135414 PMC2569852

[38] Whitfield C, Paiment A (2003) Biosynthesis and assembly of Group 1 capsular polysaccharides in *Escherichia coli* and related extracellular polysaccharides in other bacteria. Carbohydr Res 338: 2491–2502. 14670711 10.1016/j.carres.2003.08.010

[39] Baba T, Ara T, Hasegawa M, Takai Y, Okumura Y, et al (2006) Construction of *Escherichia coli* K-12 in-frame, single-gene knockout mutants: the Keio collection. Mol Syst Biol 2: 2006.0008 doi:10.1038/msb4100050 10.1038/msb4100050 PMC1681482 16738554

[40] Liu A, Tran L, Becket E, Lee K, Chinn L, et al (2010) Antibiotic sensitivity profiles determined with an *Escherichia coli* gene knockout collection: generating an antibiotic bar code. Antimicrob Agents Chemother 54: 1393–1403. 20065048 10.1128/AAC.00906-09 PMC2849384

[41] Ramstedt M, Nakao R, Wai SN, Uhlin BE, Boily J-F (2011) Monitoring surface chemical changes in the bacterial cell wall: multivariate analysis of cryo-x-ray photoelectron spectroscopy data. J Biol Chem 286: 12389–12396. 21330374 10.1074/jbc.M110.209536 PMC3069442

[42] Yethon J a, Heinrichs DE, Monteiro M a, Perry MB, Whitfield C (1998) Involvement of *waaY*, *waaQ*, and *waaP* in the modification of *Escherichia coli* lipopolysaccharide and their role in the formation of a stable outer membrane. J Biol Chem 273: 26310–26316. 9756860 10.1074/jbc.273.41.26310

[43] Lee H, Hsu F, Turk J, Eduardo A, Groisman EA (2004) The PmrA-Regulated *pmrC* Gene Mediates Phosphoethanolamine Modification of Lipid A and Polymyxin Resistance in *Salmonella enterica.* . J Bacteriol 186: 4124–4133. 15205413 10.1128/JB.186.13.4124-4133.2004 PMC421605

[44] Breazeale SD, Ribeiro A a, Raetz CRH (2003) Origin of lipid A species modified with 4-amino-4-deoxy-L-arabinose in polymyxin-resistant mutants of *Escherichia coli*. An aminotransferase (ArnB) that generates UDP-4-deoxyl-L-arabinose. J Biol Chem 278: 24731–24739. 12704196 10.1074/jbc.M304043200

[45] Fry BN, Feng S, Chen YY, Newell DG, Coloe PJ, et al (2000) The *galE* gene of *Campylobacter jejuni* is involved in lipopolysaccharide synthesis and virulence. Infect Immun 68: 2594–2601. 10768949 10.1128/iai.68.5.2594-2601.2000 PMC97464

[46] Ho TD, Waldor MK (2007) Enterohemorrhagic *Escherichia coli* O157:H7 *gal* mutants are sensitive to bacteriophage P1 and defective in intestinal colonization. Infect Immun 75: 1661–1666. 17158899 10.1128/IAI.01342-06 PMC1865682

[47] Nesper J, Lauriano CM, Klose KE, Kapfhammer D, Kraiß A, et al (2001) Characterization of *Vibrio cholerae* O1 El Tor *galU* and *galE* Mutants : Influence on Lipopolysaccharide Structure, Colonization, and Biofilm Formation. Infect Immun 69: 435–445. 11119535 10.1128/IAI.69.1.435-445.2001 PMC97901

[48] Ramos ANA, Boels IC, Willem M, Santos H (2001) Relationship between Glycolysis and Exopolysaccharide Biosynthesis in *Lactococcus lactis* . Appl Environ Microbiol 67: 33–41. 11133425 10.1128/AEM.67.1.33-41.2001 PMC92509

[49] Singh V, Satheesh S V, Raghavendra ML, Sadhale PP (2007) The key enzyme in galactose metabolism, UDP-galactose-4-epimerase, affects cell-wall integrity and morphology in *Candida albicans* even in the absence of galactose. Fungal Genet Biol 44: 563–574. 17178245 10.1016/j.fgb.2006.11.006

[50] Sakurai K, Sakaguchi T, Yamaguchi H, Iwata K (1978) Mode of action of 6-cyclohexyl-1-hydroxy-4-methyl-2(1H)-pyridone ethanolamine salt (Hoe 296). Chemotherapy 24: 68–76. 340169 10.1159/000237762

[51] Marolda CL, Lahiry P, Vines E, Saldias S, Valvano MA (2006) Micromethods for the characterization of lipid A-core and O-antigen lipopolysaccharide. Methods Mol Biol 347: 237–252. 17072014 10.1385/1-59745-167-3:237

[52] Rivera M, Bertasso A, McCaffrey C, Georgopapadakou NH (1993) Porins and lipopolysaccharide of *Escherichia coli* ATCC 25922 and isogenic rough mutants. FEMS Microbiol Lett 108: 183–187. 8387443 10.1111/j.1574-6968.1993.tb06096.x

[53] Consortium J, Microbiome H, Data P, Working G (2012) Evaluation of 16S rDNA-based community profiling for human microbiome research. PloS One 7: e39315. 22720093 10.1371/journal.pone.0039315 PMC3374619

[54] Cornelis P, Matthijs S (2002) Diversity of siderophore-mediated iron uptake systems in fluorescent pseudomonads: not only pyoverdines. Environ Microbiol 4: 787–798. 12534462 10.1046/j.1462-2920.2002.00369.x

[55] Fuse K, Fujimura S, Kikuchi T, Gomi K, Iida Y, et al (2013) Reduction of virulence factor pyocyanin production in multidrug-resistant *Pseudomonas aeruginosa* . J Infect Chemother 19: 82–88. 22865331 10.1007/s10156-012-0457-9

[56] Nordmann P, Naas T, Poirel L (2011) Global spread of Carbapenemase-producing Enterobacteriaceae. Emerg Infect Dis 17: 1791–1798. 22000347 10.3201/eid1710.110655 PMC3310682

[57] Ji SC, Wang X, Yun SH, Jeon HJ, Lee HJ, et al (2011) In vivo transcription dynamics of the galactose operon: a study on the promoter transition from P1 to P2 at onset of stationary phase. PloS One 6: e17646. 21445255 10.1371/journal.pone.0017646 PMC3060815

[58] Görke B, Vogel J (2008) Noncoding RNA control of the making and breaking of sugars. Genes Dev 22: 2914–2925. 18981470 10.1101/gad.1717808

[59] Germanier R, Fuer E (1975) Isolation and characterization of Gal E mutant Ty 21a of *Salmonella typhi*: a candidate strain for a live, oral typhoid vaccine. J Infect Dis 131: 553–558. 1092768 10.1093/infdis/131.5.553

[60] Nakao R, Senpuku H, Watanabe H (2006) *Porphyromonas gingivalis galE* is involved in lipopolysaccharide O-antigen synthesis and biofilm formation. Infect Immun 74: 6145–6153. 16954395 10.1128/IAI.00261-06 PMC1695533

[61] Görke B, Stülke J (2008) Carbon catabolite repression in bacteria: many ways to make the most out of nutrients. Nat Rev Microbiol 6: 613–624. 18628769 10.1038/nrmicro1932

[62] Fuhrer T, Fischer E, Sauer U (2005) Experimental Identification and Quantification of Glucose Metabolism in Seven Bacterial Species. J Bacteriol 187: 1581–1590. 15716428 10.1128/JB.187.5.1581-1590.2005 PMC1064017

[63] Kim HU, Kim TY, Lee SY (2010) Genome-scale metabolic network analysis and drug targeting of multi-drug resistant pathogen *Acinetobacter baumannii* AYE. Mol Biosyst 6: 339–348. 20094653 10.1039/b916446d

[64] Kamada N, Kim Y-G, Sham HP, Vallance B a, Puente JL, et al (2012) Regulated virulence controls the ability of a pathogen to compete with the gut microbiota. Science (New York, NY) 336: 1325–1329. 10.1126/science.1222195 PMC3439148 22582016

[65] Ross KL, Davis CN, Fridovich-Keil JL (2004) Differential roles of the Leloir pathway enzymes and metabolites in defining galactose sensitivity in yeast. Mol Genet Metab 83: 103–116. 15464425 10.1016/j.ymgme.2004.07.005

[66] Brown V, Sabina J, Johnston M (2009) Specialized sugar sensing in diverse fungi. Curr Biol 19: 436–441. 19249212 10.1016/j.cub.2009.01.056 PMC2762733

[67] Wasilenko J, Fridovich-Keil JL (2006) Relationship between UDP-galactose 4′-epimerase activity and galactose sensitivity in yeast. J Biol Chem 281: 8443–8449. 16452467 10.1074/jbc.M600778200

[68] Platt a, Reece RJ (1998) The yeast galactose genetic switch is mediated by the formation of a Gal4p-Gal80p-Gal3p complex. EMBO J 17: 4086–4091. 9670023 10.1093/emboj/17.14.4086 PMC1170741

[69] Bhat PJ, Murthy T V (2001) Transcriptional control of the GAL/MEL regulon of yeast *Saccharomyces cerevisiae*: mechanism of galactose-mediated signal transduction. Mol Microbiol 40: 1059–1066. 11401712 10.1046/j.1365-2958.2001.02421.x

[70] Askew C, Sellam A, Epp E, Hogues H, Mullick A, et al (2009) Transcriptional regulation of carbohydrate metabolism in the human pathogen *Candida albicans* . PLoS Pathog 5: e1000612. 19816560 10.1371/journal.ppat.1000612 PMC2749448

[71] Han T-L, Cannon RD, Villas-Bôas SG (2011) The metabolic basis of *Candida albicans* morphogenesis and quorum sensing. Fungal Genet Biol 48: 747–763. 21513811 10.1016/j.fgb.2011.04.002

[72] Braga PC, Piatti G, Conti E, Vignali F (1992) Effects of subinhibitory concentrations of ciclopirox on the adherence of *Candida albicans* to human buccal and vaginal epithelial cells. Arzneimittelforschung 42: 1368–1371. 1492854

[73] Zhou H, Shen T, Luo Y, Liu L, Chen W, et al (2010) The antitumor activity of the fungicide ciclopirox. Int J Cancer 127: 2467–2477. 20225320 10.1002/ijc.25255 PMC2888914

[74] Brockhausen I (1999) Pathways of O-glycan biosynthesis in cancer cells. Biochim Biophys Acta 1473: 67–95. 10580130 10.1016/s0304-4165(99)00170-1

[75] Baker MA, Taub RN, Whelton CH, Hindenburg A (1984) Aberrant sialylation of granulocyte membranes in chronic myelogenous leukemia. Blood 63: 1194–1197. 6585235

[76] Brockhausen I, Yang JM, Burchell J, Whitehouse C, Taylor-Papadimitriou J (1995) Mechanisms underlying aberrant glycosylation of MUC1 mucin in breast cancer cells. Eur J Biochem 233: 607–617. 7588808 10.1111/j.1432-1033.1995.607_2.x

[77] Wang X, Quinn PJ (2010) Lipopolysaccharide: Biosynthetic pathway and structure modification. Prog Lipid Res 49: 97–107. 19815028 10.1016/j.plipres.2009.06.002

[78] Gunn JS, Lim KB, Krueger J, Kim K, Guo L, et al (1998) PmrA-PmrB-regulated genes necessary for 4-aminoarabinose lipid A modification and polymyxin resistance. Mol Microbiol 27: 1171–1182. 9570402 10.1046/j.1365-2958.1998.00757.x

[79] Zhou Z, Ribeiro a a, Lin S, Cotter RJ, Miller SI, et al (2001) Lipid A modifications in polymyxin-resistant *Salmonella typhimurium*: PMRA-dependent 4-amino-4-deoxy-L-arabinose, and phosphoethanolamine incorporation. J Biol Chem 276: 43111–43121. 11535603 10.1074/jbc.M106960200

[80] Gunn JS, Ryan SS, Velkinburgh JC Van, Ernst RK, Miller SI, et al (2000) Genetic and Functional Analysis of a PmrA-PmrB-Regulated Locus Necessary for Lipopolysaccharide Modification, Antimicrobial Peptide Resistance, and Oral Virulence of *Salmonella enterica* Serovar Typhimurium Genetic. Infect Immun 68: 6139–6146. 11035717 10.1128/iai.68.11.6139-6146.2000 PMC97691

[81] Studier FW, Moffatt BA (1986) Use of bacteriophage T7 RNA polymerase to direct selective high-level expression of cloned genes. J Mol Biol 189: 113–130. 3537305 10.1016/0022-2836(86)90385-2

[82] Eisenstadt Carlton, B.C. and Brown, B.J E (1994) Gene mutation. In Gerhardt Murray, R. G. E., Wood, W. A., and Krieg, N. R. P, editors. Methods for General and Molecular Bacteriology. Washington, DC: American Society for Microbiology. 297–316.

[83] CLSI (2006) Performance standards for antimicrobial susceptibility testing: M100-S16; 16th informational supplement. Wayne, PA: Clinical and Laboratory Standards Institute.

[84] Altschul SF, Madden TL, Schaffer AA, Zhang J, Zhang Z, et al (1997) Gapped BLAST and PSI-BLAST: a new generation of protein database search programs. Nucleic Acids Res 25: 3389–3402. 9254694 10.1093/nar/25.17.3389 PMC146917

[85] Cummings L, Riley L, Black L, Souvorov A, Resenchuk S, et al (2002) Genomic BLAST: custom-defined virtual databases for complete and unfinished genomes. FEMS Microbiol Lett 216: 133–138. 12435493 10.1111/j.1574-6968.2002.tb11426.x

[86] Cole JR, Wang Q, Cardenas E, Fish J, Chai B, et al (2009) The Ribosomal Database Project: improved alignments and new tools for rRNA analysis. Nucleic Acids Res 37: D141–5. 19004872 10.1093/nar/gkn879 PMC2686447

