# Supplementary figures and images for: Toward Repurposing Ciclopirox as an Antibiotic against Drug-Resistant Acinetobacter baumannii, Escherichia coli, and Klebsiella pneumoniae
Source: PLoS One. 2013 Jul 23;8(7):e69646. doi: 10.1371/journal.pone.0069646 (PMC3720592; doi:10.1371/journal.pone.0069646)

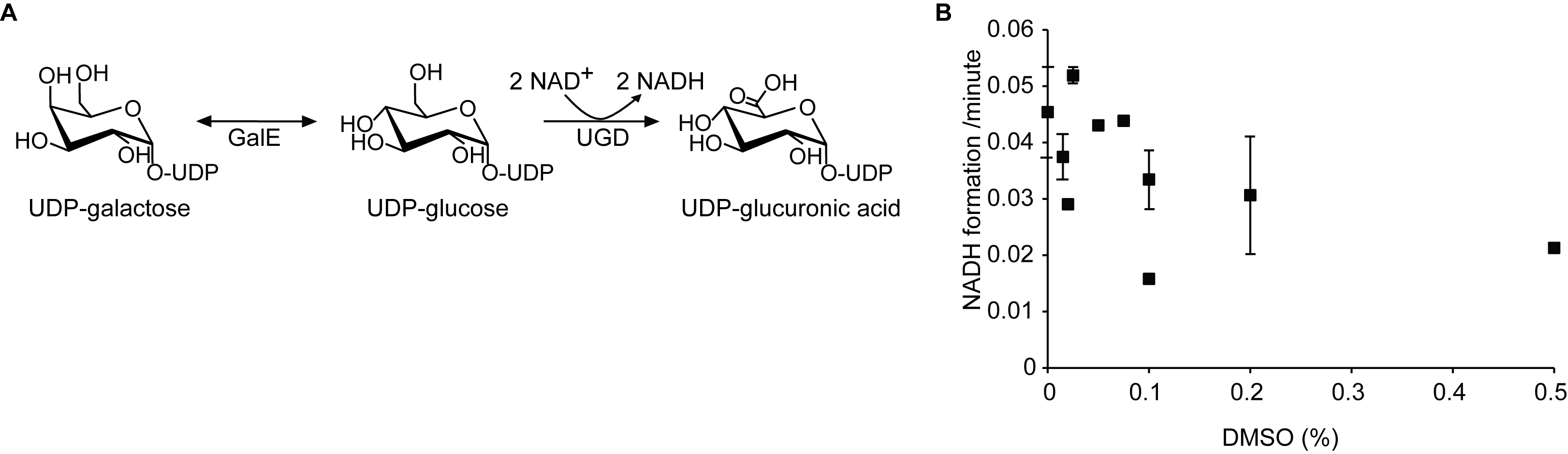

Supplement: Figure S1 — Effect of DMSO on GalE epimerization. (A) Schematic representation of GalE epimerization of UDP-galactose to UDP-glucose coupled to the activity of UDP-glucose dehydrogenase (UGD). (B) Using the assay schematized in A, the average rate of NADH formation with or without DMSO was measured three independent times per DMSO percentage. Error bars are the standard deviation from the mean. (TIF) [file pone.0069646.s001.tif]

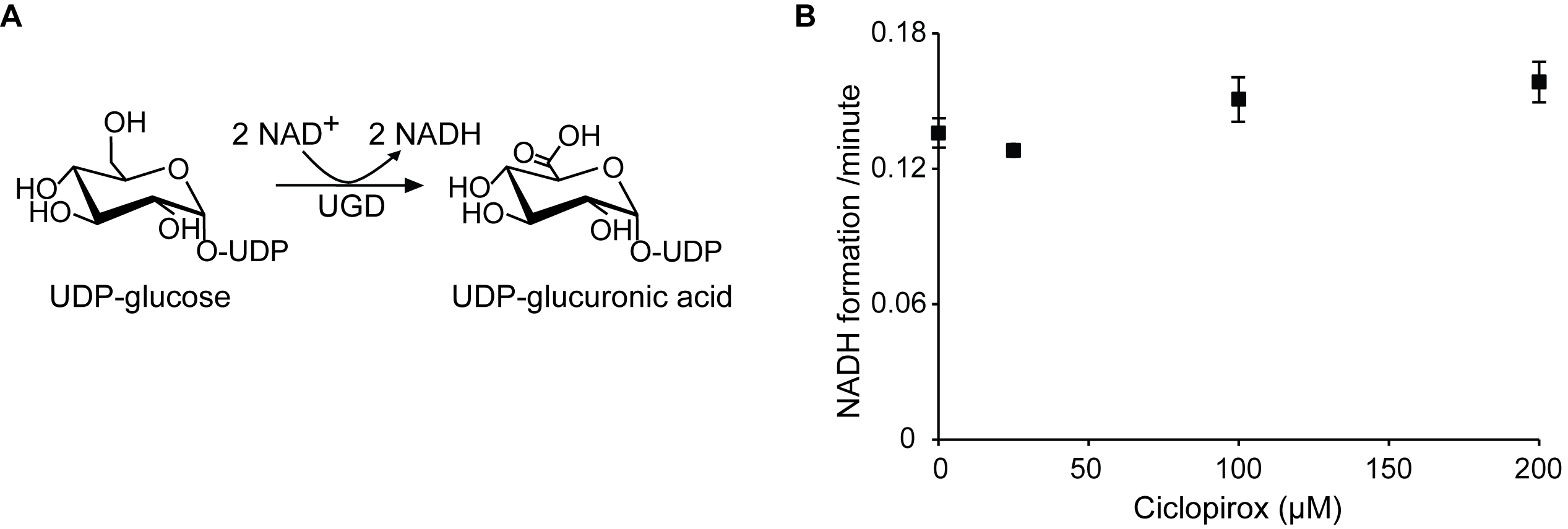

Supplement: Figure S2 — Effect of ciclopirox on UDP-glucose dehydrogenase. (A) Schematic representation of UDP-glucose dehydrogenase (UGD) activity converting UDP-glucose into UDP-glucuronic acid. (B) Using the assay schematized in A, the average rate of NADH formation with or without ciclopirox was measured three independent times per ciclopirox percentage. Error bars are the standard deviation from the mean. (TIF) [file pone.0069646.s002.tif]

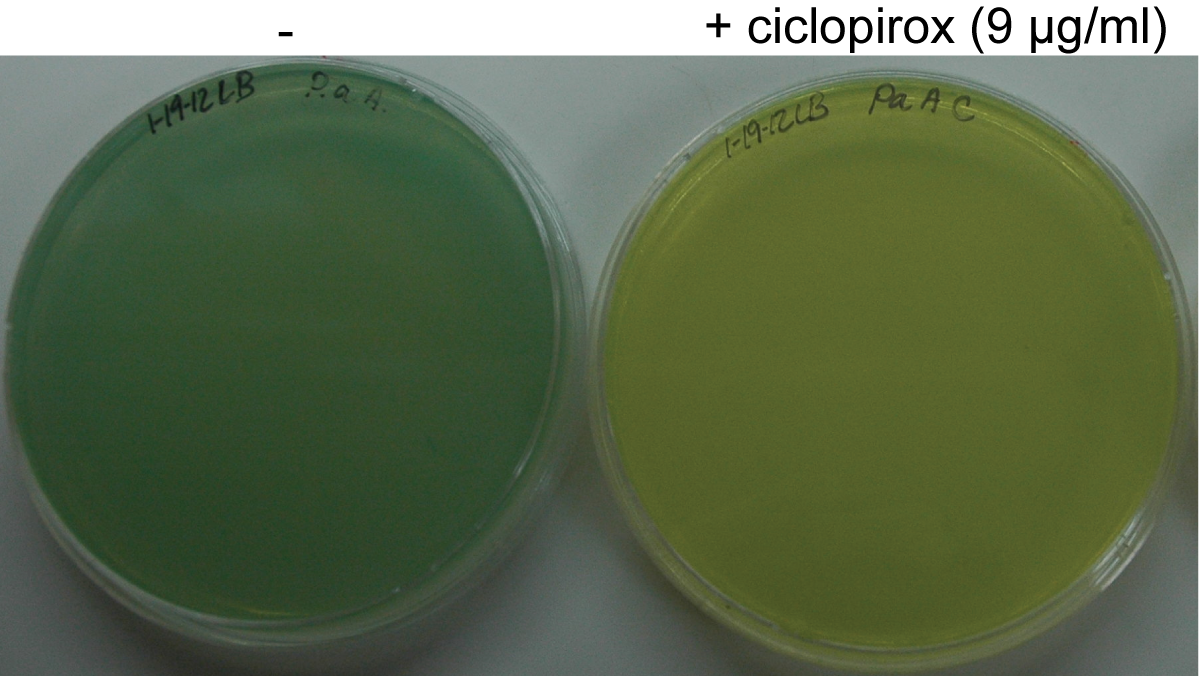

Supplement: Figure S3 — Ciclopirox effects on P. aeruginosa growth. Mid-logarithmic cultures (O.D. = 0.4) of ATCC®27853™ P. aeruginosa cultures were spread onto LB agar without (left) or with 9 µg/ml ciclopirox (middle) and grown at 37°C. After 24 hours, plates were imaged. (TIF) [file pone.0069646.s003.tif]
