# Supplementary material for: Toward Repurposing Ciclopirox as an Antibiotic against Drug-Resistant Acinetobacter baumannii, Escherichia coli, and Klebsiella pneumoniae
Source: PLoS One. 2013 Jul 23;8(7):e69646. doi: 10.1371/journal.pone.0069646 (PMC3720592; doi:10.1371/journal.pone.0069646)
Supplement: Table S1 — Antibiotic susceptibility status for fluoroquinolone-resistant clinical isolates. (DOCX) [file pone.0069646.s004.docx]

**Table S1. Antibiotic susceptibility status for fluoroquinolone-resistant clinical isolates.**

|  | Antibiotics Resisted | | | | | | |
| --- | --- | --- | --- | --- | --- | --- | --- |
| Clinical Isolate | Aminoglycoside | Nitrofurantoin | Sulfadrug | Monobactam | Penicillin | Cominbination Penicillin | Cephalosporin |
| ELZ4073 | - | - | - | - | - | - | - |
| ELZ4006 | - | - | Trimethoprim-sulfamethoxazole | - | - | - | - |
| ELZ4062 | - | - | Trimethoprim-sulfamethoxazole | - | - | - | - |
| ELZ4368 | - | - | Trimethoprim-sulfamethoxazole | - | Ampicillin | - | - |
| ELZ4277 | - | - | Trimethoprim-sulfamethoxazole | - | Ampicillin | - | - |
| ELZ4033 | Kanamycin | - | Trimethoprim-sulfamethoxazole | - | Ampicillin | - | - |
| ELZ4234 | - | Nitrofurantoin | - | - | Ampicillin | - | Cefazolin Cefepime Cefotetan Cefoxitin Ceftazidime |
| ELZ4137 | Gentamicin Tobramycin | Nitrofurantoin | - | - |  | Amoxicillin-clavulanic acid | Cefazolin Cefotetan |
| ELZ4251 | Gentamicin Tobramycin | - | Trimethoprim-sulfamethoxazole | - | Ampicillin |  | Cefazolin Cefotaxime Ceftriaxone |
| ELZ4152 | Tobramycin | Nitrofurantoin | Trimethoprim-sulfamethoxazole | - | - | Amoxicillin-clavulanic acid Ticarcillin-clavulanic acid | - |
| ELZ4054 | Gentamicin | - | Trimethoprim-sulfamethoxazole | Aztreonam | Ampicillin | - | Cefazolin Cefotaxime Cefoxitin Ceftazidime Ceftriaxone |
| ELZ4051 | Gentamicin Tobramycin | - | Trimethoprim-sulfamethoxazole | - | Ampicillin | Amoxicillin-clavulanic acid | Cefazolin Cefepime Cefotaxime Cefotetan Cefoxitin Ceftriaxone |
| ELZ4011 | Gentamicin Tobramycin | - | Trimethoprim-sulfamethoxazole | - | Ampicillin | Ticarcillin-clavulanic acid | Cefazolin Cefoxitin |
| ELZ4083 | - | Nitrofurantoin | Trimethoprim-sulfamethoxazole | - | Ampicillin | Amoxicillin-clavulanic acid Ticarcillin-clavulanic acid | Cefazolin Cefotaxime Cefotetan Cefoxitin Ceftazidime Ceftriaxone |
| ELZ4067 | - | - | Trimethoprim-sulfamethoxazole | Aztreonam | Ampicillin | Ticarcillin-clavulanic acid | Cefazolin Cefotetan Cefoxitin Ceftazidime Ceftriaxone |
| ELZ4238 | Amikacin Gentamicin Tobramycin | - | Trimethoprim-sulfamethoxazole | Aztreonam | Ampicillin | - | Cefazolin Cefotaxime Ceftazidime |
| ELZ4223 | Gentamicin Tobramycin | - | Trimethoprim-sulfamethoxazole | - | Ampicillin | Amoxicillin-clavulanic acid Ticarcillin-clavulanic acid Pipercillin-tazobactam | Cefazolin Cefoxitin |
| ELZ4004 | Gentamicin Tobramycin | - | Trimethoprim-sulfamethoxazole | Aztreonam | Ampicillin | Ticarcillin-clavulanic acid Pipercillin-tazobactam | Cefazolin Cefepime Cefotaxime Cefoxitin Ceftazidime Ceftriaxone |
| ELZ4091 | Gentamicin Tobramycin | - | Trimethoprim-sulfamethoxazole | Aztreonam | Ampicillin | Ticarcillin-clavulanic acid Pipercillin-tazobactam | Cefazolin Cefepime Cefotaxime Cefoxitin Ceftazidime Ceftriaxone |
| ELZ4220 | Gentamicin Tobramycin | Nitrofurantoin | Trimethoprim-sulfamethoxazole | - | Ampicillin | Amoxicillin-clavulanic acid Ticarcillin-clavulanic acid | Cefazolin Cefotetan Cefoxitin Cefotaxime |
| ELZ4288 | Gentamicin Tobramycin | - | Trimethoprim-sulfamethoxazole | Aztreonam | Ampicillin | Amoxicillin-clavulanic acid Ticarcillin-clavulanic acid | Cefazolin Cefotaxime Cefotetan Cefoxitin Ceftazidime Ceftriaxone |
| ELZ4240 | Amikacin Gentamicin Tobramycin | Nitrofurantoin | Trimethoprim-sulfamethoxazole | - | Ampicillin | Amoxicillin-clavulanic acid Ticarcillin-clavulanic acid Pipercillin-tazobactam | Cefazolin Cefoxitin |
| ELZ4000 | Gentamicin Tobramycin | - | Trimethoprim-sulfamethoxazole | Aztreonam | Ampicillin | Pipercillin-tazobactam | Cefazolin Cefotaxime Cefoxitin Ceftazidime Ceftriaxone |
| ELZ4013 | Gentamicin Tobramycin | Nitrofurantoin | Trimethoprim-sulfamethoxazole | Aztreonam | Ampicillin | Ticarcillin-clavulanic | Cefazolin Cefepime Cefotaxime Cefotetan Cefoxitin Ceftazidime Ceftriaxone |
| ELZ4268 | Tobramycin | Nitrofurantoin | Trimethoprim-sulfamethoxazole | Aztreonam | Ampicillin | Amoxicillin-clavulanic acid Ticarcillin-clavulanic acid Pipercillin-tazobactam | Cefazolin Cefepime Cefotamine Cefoxitin Ceftazidime Ceftriaxone |
| ELZ4486* | Amikacin Gentamicin Tobramycin | Nitrofurantoin | Trimethoprim-sulfamethoxazole | - | Ampicillin | Amoxicillin-clavulanic acid Ticarcillin-clavulanic acid Pipercillin-tazobactam | Cefazolin Cefepime Cefotaxime Cefotetan Ceftriaxone |
| ELZ4273 | Tobramycin | Nitrofurantoin | Trimethoprim-sulfamethoxazole | Aztreonam | Ampicillin | Amoxicillin-clavulanic acid Ticarcillin-clavulanic acid Pipercillin-tazobactam | Cefazolin Cefepime Cefotaxime Cefotetan Cefoxitin Ceftazidime Ceftriaxone |

* Resistant to Imipenem
