# Supplementary material for: Toward Repurposing Ciclopirox as an Antibiotic against Drug-Resistant Acinetobacter baumannii, Escherichia coli, and Klebsiella pneumoniae
Source: PLoS One. 2013 Jul 23;8(7):e69646. doi: 10.1371/journal.pone.0069646 (PMC3720592; doi:10.1371/journal.pone.0069646)
Supplement: Table S2 — Patient demographics and clinical isolate culture sites. (DOCX) [file pone.0069646.s005.docx]

**Table S2. Patient demographics and clinical isolate culture sites**

| Clinical Isolate | Age | Gender | Culture site |
| --- | --- | --- | --- |
| ATCC^®^25922™ | unknown | unknown | unknown |
| ELZ4000 | 64 | M | Blood |
| ELZ4004 | 34 | F | Urine |
| ELZ4006 | 45 | F | Urine |
| ELZ4011 | 40 | M | Urine |
| ELZ4013 | 17 | M | Urine |
| ELZ4033 | unknown | unknown | Urine |
| ELZ4045 | 21 | F | Urine |
| ELZ4046 | 32 | F | Urine |
| ELZ4051 | 46 | F | Sputum |
| ELZ4054 | 33 | F | Urine |
| ELZ4062 | 58 | F | Urine |
| ELZ4067 | 43 | F | Exudate |
| ELZ4073 | 35 | F | Urine |
| ELZ4083 | 35 | F | Urine |
| ELZ4091 | 33 | F | Urine |
| ELZ4137 | 79 | F | Urine |
| ELZ4152 | 76 | F | Urine |
| ELZ4220 | 34 | F | Urine |
| ELZ4223 | 65 | F | Urine |
| ELZ4234 | 7 | F | Urine |
| ELZ4238 | 56 | M | Kidney abscess |
| ELZ4240 | 17 | M | Urine |
| ELZ4251 | 62 | F | Urine |
| ELZ4268 | 17 | M | Urine |
| ELZ4273 | 17 | M | Urine |
| ELZ4277 | 47 | F | Urine |
| ELZ4288 | 15 | M | Urine |
| ELZ4368 | 77 | M | Urine |
| ELZ4486 | unknown | unknown | unknown |
