# Supplementary material for: Toward Repurposing Ciclopirox as an Antibiotic against Drug-Resistant Acinetobacter baumannii, Escherichia coli, and Klebsiella pneumoniae
Source: PLoS One. 2013 Jul 23;8(7):e69646. doi: 10.1371/journal.pone.0069646 (PMC3720592; doi:10.1371/journal.pone.0069646)
Supplement: Table S3 — Antibiotics MICs in galactose pathway deletion strains. (DOCX) [file pone.0069646.s006.docx]

**Table S3. Antibiotic MICs in galactose pathway deletion strains**

|  | MIC range (μg/ml) | | | |
| --- | --- | --- | --- | --- |
| Strain | Ampicillin | Azteronam | Chloramphenicol | Ciprofloxacin |
| BW25113 | 2-4 | 0.047 | 4-6 | 0.016-0.023 |
| BW25113 *∆galE* | 3 | 0.023-0.032 | 3-4 | 0.012-0.016 |
| BW25113 *∆galU* | 2-4 | 0.047 | 4-8 | 0.016 |
| BW25113 *∆rfaB* | 4 | 0.047-0.056 | 3-4 | 0.016 |
| BW25113 *∆rfaI* | 4 | 0.250 | 4 | 0.012-0.016 |
| BW25113 *∆rfaJ* | 4-6 | 0.125-0.190 | 6-8 | 0.016-0.023 |
